# Gene rearrangements in gekkonid mitochondrial genomes with shuffling, loss, and reassignment of tRNA genes

**DOI:** 10.1186/1471-2164-15-930

**Published:** 2014-10-24

**Authors:** Yoshinori Kumazawa, Saaya Miura, Chiemi Yamada, Yasuyuki Hashiguchi

**Affiliations:** Department of Information and Biological Sciences and Research Center for Biological Diversity, Graduate School of Natural Sciences, Nagoya City University, 1 Yamanohata, Mizuho-cho, Mizuho-ku, Nagoya, 467-8501 Japan; Department of Biology, Osaka Medical College, Takatsuki, Japan

**Keywords:** Gecko, Mitochondrial DNA, Gene rearrangement, tRNA, Pseudogene

## Abstract

**Background:**

Vertebrate mitochondrial genomes (mitogenomes) are 16–18 kbp double-stranded circular DNAs that encode a set of 37 genes. The arrangement of these genes and the major noncoding region is relatively conserved through evolution although gene rearrangements have been described for diverse lineages. The tandem duplication-random loss model has been invoked to explain the mechanisms of most mitochondrial gene rearrangements. Previously reported mitogenomic sequences for geckos rarely included gene rearrangements, which we explore in the present study.

**Results:**

We determined seven new mitogenomic sequences from Gekkonidae using a high-throughput sequencing method. The *Tropiocolotes tripolitanus* mitogenome involves a tandem duplication of the gene block: tRNA^Arg^, NADH dehydrogenase subunit 4L, and NADH dehydrogenase subunit 4. One of the duplicate copies for each protein-coding gene may be pseudogenized. A duplicate copy of the tRNA^Arg^ gene appears to have been converted to a tRNA^Gln^ gene by a C to T base substitution at the second anticodon position, although this gene may not be fully functional in protein synthesis. The *Stenodactylus petrii* mitogenome includes several tandem duplications of tRNA^Leu^ genes, as well as a translocation of the tRNA^Ala^ gene and a putative origin of light-strand replication within a tRNA gene cluster. Finally, the *Uroplatus fimbriatus* and *U. ebenaui* mitogenomes feature the apparent loss of the tRNA^Glu^ gene from its original position. *Uroplatus fimbriatus* appears to retain a translocated tRNA^Glu^ gene adjacent to the 5’ end of the major noncoding region.

**Conclusions:**

The present study describes several new mitochondrial gene rearrangements from Gekkonidae. The loss and reassignment of tRNA genes is not very common in vertebrate mitogenomes and our findings raise new questions as to how missing tRNAs are supplied and if the reassigned tRNA gene is fully functional. These new examples of mitochondrial gene rearrangements in geckos should broaden our understanding of the evolution of mitochondrial gene arrangements.

**Electronic supplementary material:**

The online version of this article (doi:10.1186/1471-2164-15-930) contains supplementary material, which is available to authorized users.

## Background

Metazoan mitochondrial genomes (mitogenomes) are double-stranded circular DNAs typically 16–18 kbp in size (reviewed in [1–3]). They are maternally inherited as haploid genomes with multiple copy numbers in a cell. Metazoan mitogenomes encode a set of 37 genes for two ribosomal RNAs, 22 tRNAs, and 13 respiratory protein subunits [1, 4] and also possess a major noncoding region or control region that contains signals for the initiation of replication and transcription (reviewed in [5, 6]). In addition, most vertebrate mitogenomes conserve a characteristic stem-and-loop structure between tRNA^Asn^ and tRNA^Cys^ genes that acts as the putative origin of light-strand replication (O_L_) [5].

**Figure 1:**
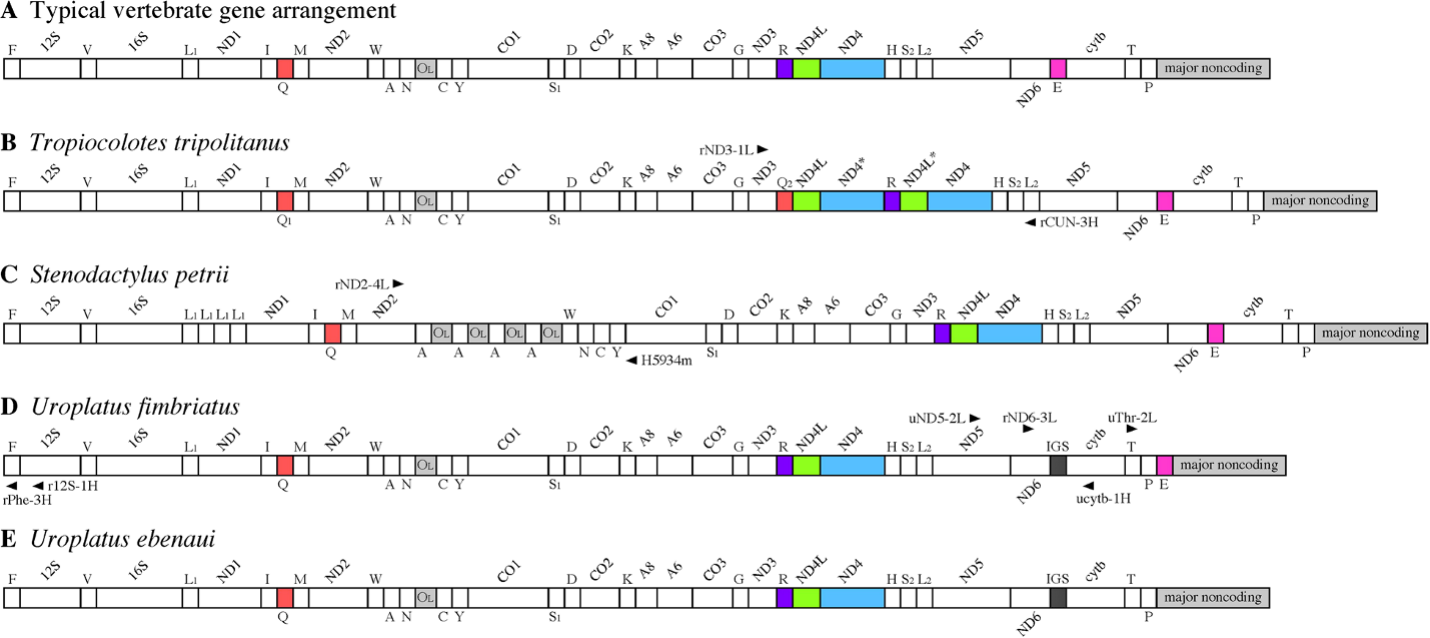
**Gene organizations of mitogenomes for (A) many vertebrates including** ***Tropiocolotes steudneri*** **,** ***Lepidodactylus lugubris***, **and** ***Phelsuma guimbeaui*** **(the typical vertebrate gene arrangement), (B)** ***Tropiocolotes tripolitanus*** **, (C)** ***Stenodactylus petrii*** **, (D)** ***Uroplatus fimbriatus*** **, and (E)** ***Uroplatus ebenaui*** **.** Circular mitogenomes are represented linearly as bars and genes encoded by the H-strand and L-strand are shown, respectively, above and below the bar. Genes with an asterisk are probable pseudogenes. Several genes relevant to our discussions on gene rearrangements are highlighted with colors. For gene names, ND1-6 and 4L represent NADH dehydrogenase subunits 1-6 and 4L. CO1-3 stand for cytochrome oxidase subunit 1-3. cytb, A6 and A8 represent cytochrome *b*, ATPase subunit 6 and 8, respectively. 12S and 16S stand for 12S rRNA and 16S rRNA, respectively. Transfer RNA genes are depicted with the corresponding single-letter amino acid and, in *T. tripolitanus*, two glutamine tRNA genes are discriminated by Q_1_ and Q_2_. L_1_ and L_2_ represent tRNA^Leu^(UUR) and tRNA^Leu^(CUN) genes, respectively, and S_1_ and S_2_ represent tRNA^Ser^(UCN) and tRNA^Ser^(AGY) genes, respectively. O_L_ represents the putative L-strand replication origin. IGS stands for an intergenic sequence in the ND6/cytb gene boundary (see text). The position and orientation of several PCR primers (see Additional file 1: Table S1 for their sequences) that were used to amplify and sequence the rearrangement-related regions are also shown.

The organization of the 37 genes and the major noncoding region varies considerably between metazoan classes but is relatively conserved within Vertebrata [2]. The typical vertebrate gene organization (Figure 1A), which was first revealed for the human mitogenome [4], is shared by many species of fishes, amphibians, reptiles, and mammals. However, deviation from this typical organization has been found in species of all these vertebrate groups and birds ([2, 3, 7, 8] and refs. therein). The majority of gene rearrangement cases in vertebrate mitochondrial genomes involve shuffling of some neighboring genes (most typically clustered tRNA genes) or the translocation of genes across duplicated control regions, for example in snake mitogenomes [2, 9]. Gene inversions are quite rare in vertebrate mitogenomes though not unknown [10].

Recent technical advances in sequencing have led to the rapid accumulation of complete or nearly complete mitogenomic sequences and the mitogenomic sequences of over 1800 vertebrate species are currently known [3]. Approximately half of them are from fishes with the remainder from tetrapods, where mammals dominate over reptiles. To the best of our knowledge, only 13 species have been sequenced from the Gekkota, which consists of more than 1500 species of geckos and their allies [11]. These known gekkotan mitogenomes share the typical vertebrate gene organization although polymorphic tandem duplications with varying sizes (6–9 kbp) have been reported for the parthenogenetic *Heteronotia binoei* [12] and potential pseudogenization of the tRNA^Gln^ gene was found in *Hemitheconyx caudicinctus* [13].

Previous studies proposed that some vertebrate groups may be more susceptible to mitogenomic gene rearrangements than the others. For example, ranoid frogs include a variety of gene rearrangements in their mitogenomes while most non-neobatrachian frogs conserve the typical gene organization ([8, 14] and refs. therein). Amongst lizards, Agamidae contain several different types of gene rearrangement [7, 10, 15, 16] but no mitogenomic rearrangement has been reported from the closely related family, Iguanidae [17]. This heterogeneity in the occurrence of gene rearrangements among different vertebrate groups has been examined in relation to loss of the light-strand replication origin [15, 18], duplication of the control region [2, 19], or changes of the rate of molecular evolution [20], although none of these causal hypotheses have been fully examined across diverse vertebrate groups.

Here, we report seven new mitogenomic sequences from Gekkonidae (Reptilia; Squamata) and describe several new gene rearrangements that involve shuffling, loss, and reassignment of tRNA genes. We discuss evolutionary mechanisms for the gene rearrangements and their effects on the mitochondrial translational system.

## Results

### Gene arrangement in the *Tropiocolotes tripolitanus*mitogenome

We used high-throughput sequencing to determine nucleotide sequences of seven new mitochondrial genomes from Gekkonidae (see Table 1 for scientific names and accompanying information of the seven geckos). Although mitogenomic sequences of *Tropiocolotes steudneri*, *Lepidodactylus lugubris*, and *Phelsuma guimbeaui* were found to possess the typical vertebrate gene organization (Figure 1A), deviations from this organization were seen in the other gekkonid mitogenomes.

**Table 1:** **Gekkonid mitogenomic sequences newly determined in this study**

Scientific name	Common name	mtDNA size (bp)	Accession no.	Voucher no.
*Tropiocolotes tripolitanus*	Northern sand gecko	20248	AB661661	SDNCU-A1648
*Tropiocolotes steudneri*	Algerian sand gecko	15863*	AB738944	SDNCU-A1652
*Stenodactylus petrii*	Anderson's short-fingered gecko	18672	AB738952	SDNCU-A1654
*Lepidodactylus lugubris*	Mourning gecko	16762	AB738945	SDNCU-A1650
*Phelsuma guimbeaui*	Orange-spotted day gecko	17533	AB661664	SDNCU-A1651
*Uroplatus fimbriatus*	Common flat-tail gecko	16780	AB612276	SDNCU-A1649
*Uroplatus ebenaui*	Nosy be flat-tail gecko	16830	AB738950	SDNCU-A1653

Accession and voucher numbers represent, respectively, numbers of mitogenomic nucleotide sequences registered to the DDBJ/EMBL/GenBank database and those of whole-body specimens registered to SDNCU (the Specimen Depository of the Graduate School of Natural Sciences, Nagoya City University).

An asterisk means that the corresponding mitogenome could not be completely sequenced because of extensive long tandem duplications in the major noncoding region.

The complete mitochondrial genome sequence of *Tropiocolotes tripolitanus* is 20,248 bp (Table 1) and encodes all 37 mitochondrial genes in addition to containing the major noncoding region (2,923 bp) located between tRNA^Pro^ and tRNA^Phe^ genes (Figure 1B). At the 5’ end of the major noncoding region, there are four tandem repeats of a 74-bp sequence, while at its 3’ end there are 10 tandem repeats of a 100-bp sequence, followed by a second weakly repetitive sequence. In the middle of the major noncoding region, three conserved sequence block (CSB) motifs (CSB-1, CSB-2, and CSB-3) [21] are found. The central part of the major noncoding region is therefore regarded as the control region that regulates replication and transcription of the mitochondrial genome [5].

The *T. tripolitanus* mitogenome has the typical vertebrate gene organization, except for a region between NADH dehydrogenase subunit 3 (ND3) and tRNA^His^ genes (Figure 1B). This region usually contains an array of genes (ND3, tRNA^Arg^, ND4L, ND4, and tRNA^His^) in the typical vertebrate organization (Figure 1A). However, the corresponding region in *T. tripolitanus* contains a rearranged set of genes (ND3, tRNA^Gln^, ND4L, ND4*, tRNA^Arg^, ND4L*, ND4, and tRNA^His^) where genes with an asterisk may be pseudogenes (see below for details). To exclude the possibility that this gene arrangement resulted from erroneous high-throughput DNA sequencing or assembly, we carefully amplified and re-sequenced this region using various combinations of 10 species-specific primers (Ttri-4L to Ttri-8L and Ttri-4H to Ttri-8H; see Additional file 1: Table S1 and Figure S1). The resultant sequence was identical to the one determined by high-throughput DNA sequencing.

**Figure 2:**
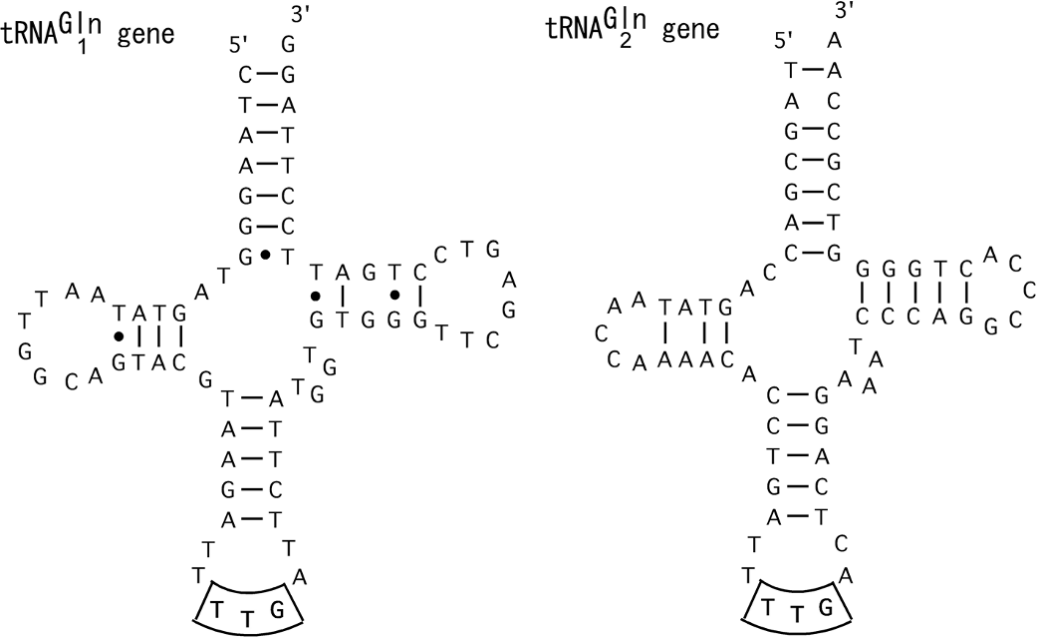
**Secondary structures of two tRNA** ^**Gln**^ **genes encoded in the** ***T. tripolitanus*** **mitogenome.** tRNA^Gln^_1_ and tRNA^Gln^_2_ genes correspond to Q_1_ and Q_2_ genes of Figure 1B, respectively. Watson-Crick and wobble base pairs are shown with a bar and a dot, respectively. Anticodon sequence for the glutamine tRNA gene (TTG) is highlighted.

**Figure 3:**
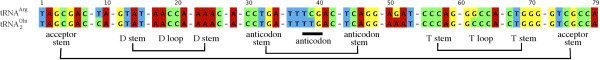
**Sequence similarity of** ***T. tripolitanus*** **tRNA** ^**Arg**^ **and tRNA** ^**Gln**^_**2**_ **genes.** Sense-strand sequences for the *T. tripolitanus* tRNA^Arg^ and tRNA^Gln^_2_ genes are aligned. The tRNA gene sequences are divided into structural elements, such as the D loop and acceptor stem, and three nucleotides corresponding to the anticodon are highlighted.

The tRNA^Gln^_2_ gene next to the ND3 gene (denoted Q_2_ in Figure 1B) is encoded by the heavy strand, while another tRNA^Gln^_1_ gene (Q_1_ in Figure 1B) encoded by the light strand occurs between tRNA^Ile^ and tRNA^Met^ genes. The position and orientation of the latter tRNA^Gln^_1_ gene matches the typical vertebrate gene organization (Figure 1A). Figure 2 illustrates the secondary structures of these two tRNA^Gln^ genes. Both the tRNA^Gln^ genes can assume stable clover-leaf structures. The tRNA^Gln^_2_ gene has a clear sequence similarity with the tRNA^Arg^ gene (Figure 3); there are only four base differences between them with one at the second anticodon position (T for the tRNA^Gln^_2_ gene and C for the tRNA^Arg^ gene). These two tRNA genes are thus paralogs and one of them (the tRNA^Gln^_2_ gene; see below) was created by gene duplication and subsequent base substitution at the second anticodon position.

Regions adjacent to the tRNA^Gln^_2_ and tRNA^Arg^ genes are homologous, sharing sequences related to ND4L genes (see Additional file 2: Figure S2). The former has a complete ND4L coding region, whereas the latter has frequent indels and severely reduced sequence similarity to ND4L genes from other geckos (Additional file 2: Figure S2). In-frame translation of this ND4L pseudogene does not show any detectable level of sequence similarity with ND4L amino acid sequences of other geckos due to frameshift indels (Additional file 2: Figure S3).

These two ND4L-related regions are followed by another pair of homologous sequences with high sequence similarity to ND4 genes from other geckos (Additional file 2: Figure S4). These two ND4-related sequences do not have in-frame stop codons and are easily aligned to each other without indels (Additional file 2: Figure S4). The second ND4-related sequence preceding the tRNA^His^ gene is shorter than the first one preceding the tRNA^Arg^ gene but has a slightly increased sequence similarity to ND4 sequences from other geckos, especially at amino acids 291–330 and 403–426 (Additional file 2: Figure S5). We therefore tentatively assume the second sequence to be a legitimate ND4 gene and regard the first one as a possible pseudogene. However, it also seems possible that both copies are functional genes in *T. tripolitanus* mitochondria.

### Gene arrangement in the *Stenodactylus petrii*mitogenome

The *S. petrii* mitogenome is 18,672 bp in length (Table 1) and includes all 37 mitochondrial genes (Figure 1C). It shows two changes from the typical vertebrate gene organization. First, there are four tandemly duplicated copies of tRNA^Leu^ (UUR) between 16S rRNA and ND1 genes. These four genes have high sequence similarity to each other (Additional file 1: Figure S6) and it is evident that they have been created by recent tandem duplications. The first gene located at the 5’ end of this tandem duplication lacks some basic tRNA secondary structures and may now be a pseudogene. The fourth copy seems to have the most stable secondary structure but the second and third copies may also be functional tRNA^Leu^ (UUR) genes in light of the structural criterion of mitochondrial tRNA genes [22]. We have samples of two more *S. petrii* individuals. Sequencing the corresponding region of these individuals showed a single tRNA^Leu^ (UUR) gene between 16S rRNA and ND1 genes (data not shown).

Second, there is a shuffling of tRNA genes and the O_L_ contained in the WAN (O_L_) CY tRNA gene cluster. Four tandem copies of tRNA^Ala^ and the O_L_ were found at the 5’ end of the remaining WNCY genes (Figure 1C). All four tRNA^Ala^ and O_L_ copies have an identical sequence (data not shown), suggesting that the tandem duplications were very recent. We amplified and sequenced a mitogenomic region between the ND2 and cytochrome oxidase subunit 1 (CO1) genes from the two additional *S. petrii* individuals to show that one has only two tandem repeats of tRNA^Ala^ and O_L_ while the other has four tandem repeats (see Additional file 3: Figure S7 for sizes of the amplified products and Additional file 4: Table S2 for accession numbers of these sequences with which annotation details can be referred to). Repeat number of this region is polymorphic within species.

### Occurrence of gene rearrangements in other *Tropiocolotes*and *Stenodactylus*species

The genus *Tropiocolotes* includes 10 species distributed in Saharo-Arabian regions [11]. Another genus, *Stenodactylus*, with a similar distribution, is closely related to *Tropiocolotes* according to recent molecular phylogenetic studies [23–26], although precise phylogeny at the species level has not been established. We examined the occurrence of the gene rearrangements found in *T. tripolitanus* and *S. petrii* mitogenomes among other species of these genera. First, the *Tropiocolotes steudneri* mitogenomic sequence is 15,863 bp in length (Table 1). The major noncoding region of this species contains rather long arrays of repetitive sequences that were not completely sequenced. The *T. steudneri* mitogenome includes all sets of 37 mitochondrial genes with the typical gene organization of vertebrates (Figure 1A). Neither of the two types of gene rearrangements found in *T. tripolitanus* and *S. petrii* occur in this species.

We also examined gene organizations of a few other species by PCR amplification and sequencing. Figure S8 in Additional file 3 shows 1% agarose gel electrophoresis of PCR products amplified using rND3-1L and rCUN-3H primers (see Additional file 1: Table S1 for primer sequences and Figure 1B for their locations). As expected, a large product was amplified from *T. tripolitanus* (~4.2 kbp: lane 1) owing to the gene rearrangements described above. In contrast, *Microgecko* (recently moved from *Tropiocolotes*) *persicus*, *Tropiocolotes steudneri*, and *Stenodactylus petrii* gave rise to shorter products (~2.2 kbp: lanes 2–4), supporting that the mitogenomes of these species do not have the above gene rearrangements.

These results suggest that the rearranged gene arrangements shown in Figure 1B are not widely distributed among *Tropiocolotes* and *Stenodactylus* geckos. The gene rearrangements may have occurred relatively recently on a lineage leading to *T. tripolitanus* after its divergence from the other examined species. This view is supported by an observation that two paralogous ND4 amino acid sequences of *T. tripolitanus* are much more similar to each other than they are to counterparts in other gecko species (Additional file 2: Figure S5). These two paralogous sequences also share an insertion at sites 253–261 (Additional file 2: Figure S5), suggesting that this insertion event took place after the divergence from a lineage leading to *T. steudneri* but before the duplication of the ND4 gene.

The mitogenomic region between the ND2 and CO1 genes amplified from *Stenodactylus doriae* was somewhat longer than that from *Stenodactylus slevini* (Additional file 3: Figure S7). *Stenodactylus slevini* turned out to have the typical WAN(O_L_)CY gene organization but *S. doriae* had another unique gene arrangement: WAN*(O_L_)CNY, where N* represents a possible pseudogene of the tRNA^Asn^ gene (see Additional file 4: Table S2 for accession numbers of nucleotide sequences deposited with complete annotation). The N* gene has a considerably weaker acceptor-stem secondary structure than the N gene (data not shown). Together with the information derived from *T. tripolitanus* and *T. steudneri* (Figure 1), these results suggest that the gene rearrangement found in *S. petrii*, in which tRNA^Ala^ and O_L_ are translocated to the 5’ end of WNCY genes (Figure 1C), is not widely distributed among *Tropiocolotes* and *Stenodactylus* geckos. This translocation and the possible translocation of the tRNA^Asn^ gene found in *S. doriae* probably took place independently in each lineage.

### Loss of the tRNA^Glu^gene from the *Uroplatus ebenaui*mitogenome

**Figure 4:**
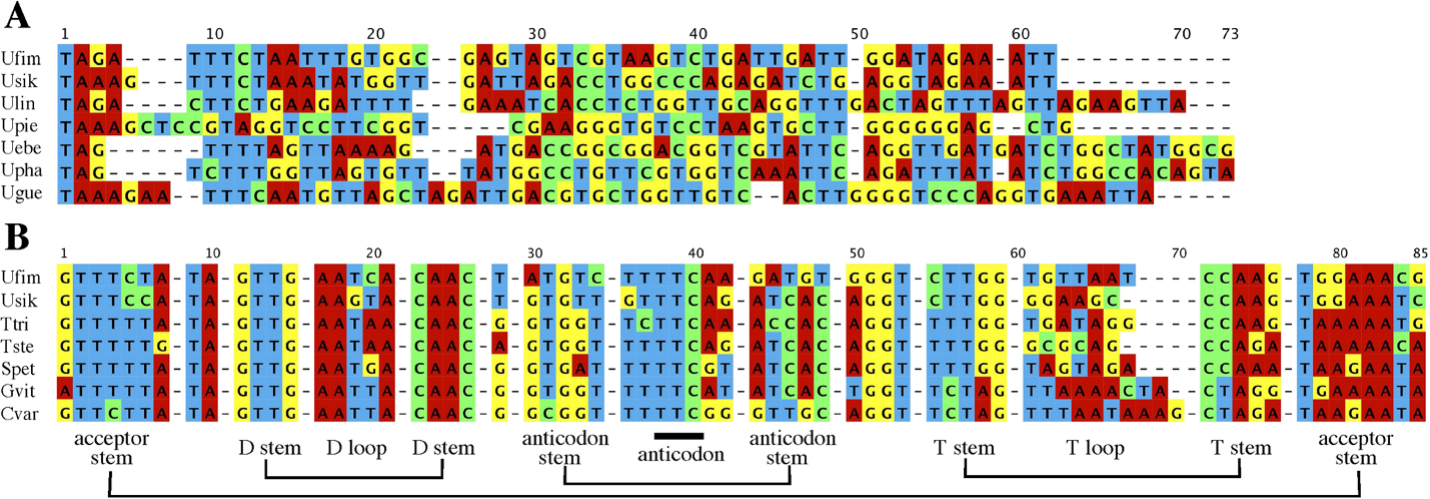
**Heavy-strand nucleotide sequences between ND6 and cytb genes for** ***Uroplatus*** **(A) and other geckos (B).** Sequences in this region for non-*Uroplatus* geckos represent tRNA^Glu^ genes, which are aligned based on clover-leaf secondary structure [22]. In **B**, tRNA^Glu^ gene sequences found at the 5’ end of the major noncoding region for *U. fimbriatus* and *U. sikorae* are also shown. Alignment of the *Uroplatus* intergenic sequences in **A** was made with the aid of ClustalX [27]. *Uroplatus* gecko sequences were given the following abbreviations: Ufim, *U. fimbriatus*; Usik, *U. sikorae*; Ulin. *U. lineatus*; Upie, *U. pietschmanni*; Uebe, *U. ebenaui*; Upha, *U. phantasticus*; and Ugue, *U. guentheri* (see Additional file 4: Table S2 for accession numbers). Sequence data of tRNA^Glu^ gene sequences for non-*Uroplatus* geckos are taken from *Tropiocolotes tripolitanus* (Ttri; this study), *Tropiocolotes steudneri* (Tste; this study), *Stenodactylus petrii* (Spet; this study), *Gekko vittatus* (Gvit; accession No. AB178897), and *Coleonyx variegatus* (Cvar; AB114446).

Complete mitogenomic sequences obtained for *Uroplatus fimbriatus* and *U. ebenaui* are 16,780 and 16,830 bp in length, respectively (Table 1). These mitogenomes possess the typical vertebrate gene organization, except for the disappearance of tRNA^Glu^, which is usually located between ND6 and cytochrome *b* (cytb) genes (Figures 1D and E). The corresponding intergenic region retains 54- and 62-bp sequences in each species (Figure 4A). However, these sequences do not show detectable sequence similarity to tRNA^Glu^ genes from five non-*Uroplatus* geckos (Figure 4B). The tRNA^Glu^ genes of these non-*Uroplatus* geckos are 68–72 bp in length, somewhat longer than the *Uroplatus* intergenic sequences.

We sequenced this intergenic region for five more *Uroplatus* species (*U. pietschmanni*, *U. sikorae*, *U. guentheri*, *U. phantasticus*, and *U. lineatus*) with rND6-3L (or uND5-2L) and ucytb-1H primers (see Figure 1D and Additional file 1: Table S1 for primer positions and sequences, respectively). Intergenic sequences of 55–64 bp in length were found in each species but they do not show appreciable sequence similarity with each other (Figure 4A). Thus, it is unlikely that these intergenic sequences in *Uroplatus* species encode any conserved gene sequence. There is also no evidence to suggest that these intergenic sequences were evolutionarily derived from tRNA^Glu^ genes.

Functional tRNA^Glu^ gene sequences were carefully searched for over the complete mitogenomic sequences of the two *Uroplatus* taxa. It was found that the *U. fimbriatus* mitogenome encodes a tRNA^Glu^ gene adjacent to the 5’ end of the major noncoding region (Figure 1D). However, no tRNA^Glu^-like sequence was found in the *U. ebenaui* mitogenome (Figure 1E). Coding regions in both taxa between tRNA^Phe^ and tRNA^Pro^ genes do not have a notable intergenic region >50 bp in length, except for the ND6-cytb intergenic region described above.

The major noncoding regions of *U. fimbriatus* and *U. ebenaui* are 1,344 bp and 1,456 bp, respectively. These noncoding regions include tandem repeat sequences and the CSB1-3 sequences but tRNA^Glu^-like structures were not found, either by the COVE program as implemented in DOGMA [28] or by visual inspection for a standard mitochondrial tRNA gene structure [22]. We therefore conclude that the tRNA^Glu^ gene is lacking from the mitogenome of *U. ebenaui*.

The major noncoding region was amplified and sequenced for the other five *Uroplatus* species using uThr-2L and r12S-1H (or rPhe-3H) primers (see Figure 1D and Additional file 1: Table S1 for primer positions and sequences, respectively). As a result, only one of them (*U. sikorae*) has the tRNA^Glu^ gene at the 5’ end of the major noncoding region, similar to *U. fimbriatus*, whereas *U. pietschmanni*, *U. guentheri*, *U. phantasticus*, and *U. lineatus* do not have the tRNA^Glu^ gene located near the major noncoding region, as in *U. ebenaui* (see Additional file 4: Table S2 for accession numbers of determined sequences). These results show that the disappearance of the tRNA^Glu^ gene from the ND6/cytb junction is a common feature among the *Uroplatus* mitogenomes but that translocation to the 5’ end of the major noncoding region only occurs in some *Uroplatus* species.

## Discussion

### Mechanism of gene rearrangements

**Figure 5:**
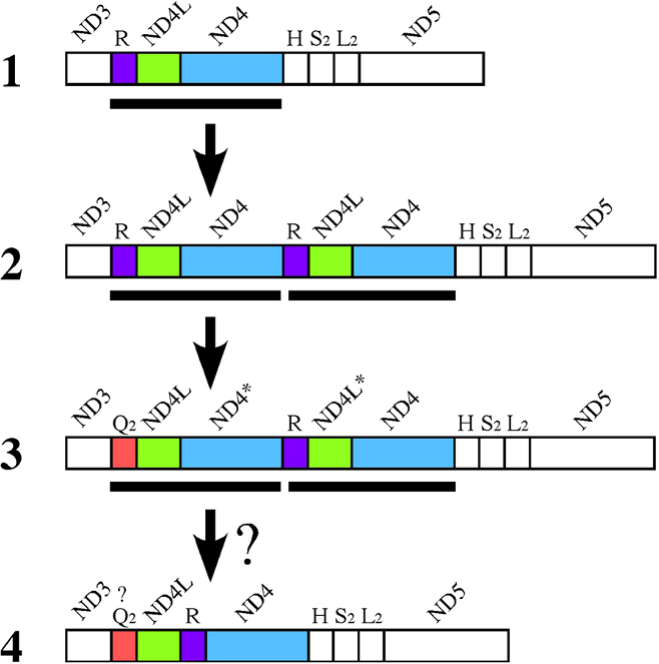
**Plausible pathway of the gene rearrangements found for the** ***T. tripolitanus*** **mitogenome based on the tandem duplication-random loss model.** Mitochondrial genes are illustrated as in Figure 1 and thick horizontal bars show a unit for tandem duplication. From the typical gene arrangement **(state 1)**, three genes were tandemly duplicated **(state 2)**. Reassignment of a tRNA gene (R to Q) and pseudogenization of duplicate protein genes gave rise to the *T. tripolitanus* gene arrangement **(state 3)**. In future, deletion of redundant genes or pseudogenes may lead to a rearranged organization shown as **state 4**.

In the *T. tripolitanus* mitogenome (Figure 1B), a region from the tRNA^Gln^_2_ gene to the ND4* gene has a sequence similarity with the region from the tRNA^Arg^ gene to the ND4 gene. This gene rearrangement originated from the tandem duplication of three genes: tRNA^Arg^, ND4L and ND4 (Figure 5). One duplicate copy of the ND4L and ND4 genes has subsequently been pseudogenized, while in a duplicate copy of tRNA^Arg^ a base substitution (C to T) at the second anticodon position converted the identity of the tRNA gene from tRNA^Arg^ to tRNA^Gln^. Three accompanying base substitutions, at positions between the acceptor and D stems, in the extra arm and in the T loop, have also occurred (Figure 3).

This plausible mechanism for the gene rearrangement that gave rise to the *T. tripolitanus* mitogenome (Figure 5) is consistent with the tandem duplication-random loss (TDRL) model [29] that has been postulated to explain most vertebrate mitochondrial gene rearrangements [2]. The TDRL model assumes a tandem duplication of a mitochondrial DNA segment and subsequent deletion of one of the duplicate gene copies, leading to a rearranged gene organization or reversal to the original organization. Deletion of the redundant gene copy may happen rapidly as it is free from functional constraint and therefore base changes can readily occur, facilitating its pseudogenization or complete deletion. This may also be driven by strong pressure for size reduction of metazoan mitochondrial genomes [1, 30]. The finding of duplicate genes between tRNA^Gln^_2_ and ND4 genes in the *T. tripolitanus* mitogenome, but not in any other mitogenomes of closely related species (Additional file 3: Figure S8), is in agreement with this reasoning if the duplication is recent. Thus, we consider that the gene organization found in the *T. tripolitanus* mitogenome is not stable and may soon lead to the complete deletion of redundant pseudogenes, as shown in Figure 5. Alternatively, the apparently redundant ND4L and ND4 pseudogenes may not be deleted easily if they play roles in translating mRNAs for overlapping protein genes (i.e., ND4L preceding ND4* and ND4 next to ND4L*). It is well known that reading frames for vertebrate ND4L and ND4 genes partly overlap and that mature mRNAs for these genes occur as a di-cistronic mRNA [4].

With respect to the mechanisms underlying the gene rearrangements in the *Stenodactylus petrii* mitogenome (Figure 1C), tandem duplications of the tRNA^Leu^(UUR) gene can occur by slipped-strand mispairing during mitogenome replication [31]. In the WAN (O_L_)CY tRNA gene cluster, translocation of the tRNA^Ala^ gene and O_L_ (i.e., from WAN (O_L_)CY to A(O_L_)WNCY) probably occurred first by a process consistent with the TDRL model [29]. Then, the slipped-strand mispairing could have resulted in 4-fold copying of A(O_L_) to generate the *S. petrii* gene arrangement (Figure 1C). The intraspecific occurrence of 2- and 4-fold copies of the A(O_L_), as described in Results, are consistent with this mechanism.

We also inferred the process of loss and translocation of the tRNA^Glu^ gene for *Uroplatus* geckos. Because all *Uroplatus* taxa examined in this study lack a tRNA^Glu^ gene at the ND6/cytb gene boundary and because all non-*Uroplatus* geckos examined to date have this gene at this location, the disappearance of the tRNA^Glu^ gene from this boundary likely occurred in the common ancestor of *Uroplatus*. The most straightforward explanation is that the tRNA^Glu^ gene was translocated to the 5’ end of the major noncoding region by the TDRL of a four gene block: tRNA^Glu^, cytb, tRNA^Thr^, and tRNA^Pro^ (the status seen for *U. fimbriatus* and *U. sikorae* mitogenomes; Figure 1D). This translocated tRNA^Glu^ gene was later lost from all other *Uroplatus* species, giving rise to the gene arrangement shown in Figure 1E.

Previous molecular phylogenetic studies of *Uroplatus* [32, 33] suggested a sister relationship between *U. fimbriatus* and *U. sikorae*; however, they are not sister species but are nested in a clade of other *Uroplatus* species (i.e., *U. guentheri*, *U. ebenaui*, *U. phantasticus*, *U. pietschmanni*, and *U. lineatus*). If true, this raises the possibility that the mitogenome of the most recent common ancestor of all *Uroplatus* taxa had the tRNA^Glu^ gene at the 5’ end of the major noncoding region and that it has disappeared from descendant *Uroplatus* lineages multiple times. An alternative possibility is that the tRNA^Glu^ gene was already lost from the most recent common ancestor but that a tRNA^Glu^ gene was newly created in the common ancestor of *U. fimbriatus* and *U. sikorae* by a mechanism such as tandem duplication of another tRNA gene and reassignment of a duplicate gene copy to tRNA^Glu^ via anticodon mutation. However, the tRNA^Glu^ genes of *U. fimbriatus* and *U. sikorae* retain high sequence similarity to those of other geckos (Figure 4B), which is not consistent with the latter possibility.

### Two functional tRNA^Gln^genes in the *T. tripolitanus*mitogenome?

An intriguing question is whether the *T. tripolitanus* mitogenome encodes two functional tRNA^Gln^ genes whose products can function in mitochondrial protein synthesis. CAA and CAG are two glutamine codons in the genetic code of vertebrate mitochondria and these codons are usually decoded by a single tRNA^Gln^ encoded in a mitogenome [1, 4]. There is no need to duplicate the tRNA^Gln^ gene for mitochondrial protein synthesis. Table S3 in Additional file 4 provides evidence for no genetic code change at these codons in *T. tripolitanus* mitochondria. There is also no evidence for codon use change. Glutamine codons (CAA + CAG) appear in protein-coding genes of the *T. tripolitanus* mitogenome as frequently as in the mitogenomes of 17 other geckos (Table 2). The relative frequency of CAA vs. CAG codons is not significantly different from that averaged among the 17 geckos (Table 2).

**Table 2:** **Codon usage at codons for glutamine and glutamic acid**

Taxon	Codons for Gln			Codons for Glu			Number of all codons ^1^
	CAA	CAG	CAA + CAG	GAA	GAG	GAA + GAG	
*Tropiocolotes tripolitanus*	67	13	80	55	18	73	3447
*Tropiocolotes steudneri*	75	10	85	61	11	72	3436
*Uroplatus fimbriatus*	64	18	82	55	18	73	3442
*Uroplatus ebenaui*	65	14	79	42*	31*	73	3441
17 geckos^2^	69.5	12.0	81.5	58.9	16.8	75.7	3441.0

^1^Number of all codons is a sum of all codons that appear in alignable regions of 13 protein-coding genes for each taxon.

^2^Values for 17 geckos are averaged among these taxa. They include *Teratoscincus keyserlingii* (accession No. AY753545), *Hemidactylus frenatus* (GQ245970), *Gekko swinhonis* (JQ906550), *Gekko gecko* (AY282753), *Gekko vittatus* (AB178897), *Heteronotia binoei* (EF626807), *Tarentola mauritanica* (EU443255), *Coleonyx variegatus* (AB114446), *Hemitheconyx taylori* (AB610503), and *Hemitheconyx caudicinctus* (AB610502) in addition to 7 taxa listed in Table 1.

*Values with an asterisk mean that relative frequency of codons for CAA vs. CAG, GAA vs. GAG, or CAA + CAG vs. GAA + GAG is significantly (*p* < 0.05) different from that averaged among 17 geckos.

The secondary structures of the tRNA^Gln^_1_ and tRNA^Gln^_2_ genes (Figure 2) conserve several features of functional mitochondrial tRNA genes [22]. Briefly, both the tRNA^Gln^ genes retain many base pairings in the stem regions and share an identical anticodon sequence (TTG) in the middle of a canonical 7-nucleotide anticodon-loop. The 5’ and 3’ nucleotides of the anticodon are, respectively, T and a purine (G) for both genes. Two intervening nucleotides occur between the acceptor-stem and D-stem, whereas a single extra nucleotide occurs between the D-stem and anticodon-stem. The extra arm between the anticodon-stem and T-stem has four nucleotides in both tRNA^Gln^ genes, the typical number for vertebrate mitochondrial tRNA genes (3–5 nucleotides) [22]. Finally, no intervening nucleotide occurs between the T-stem and acceptor-stem. These tRNA^Gln^ genes appear to comply with the basic structural requirements of mitochondrial tRNA genes.

However, an extra requirement should be considered for tRNA^Gln^ genes encoded by vertebrate mitogenomes. Eukaryotic mitochondria, as well as all known archaea and most bacteria, lack a glutaminyl-tRNA synthetase (GlnRS), which is responsible for charging tRNA^Gln^ with glutamine [34, 35]. Instead, they use the non-discriminating glutamyl-tRNA synthetase (GluRS) to charge both tRNA^Glu^ and tRNA^Gln^ with glutamic acid, thus forming Glu-tRNA^Glu^ and Glu-tRNA^Gln^, respectively. Glu-tRNA^Gln^ is then converted to Gln-tRNA^Gln^ by an amidotransferase (AdT) (reviewed in [36]). The crystal structure of an archaeal non-discriminating GluRS in comparison with that of an *E. coli* GlnRS-tRNA^Gln^ complex [37] indicated that the non-discriminating GluRS recognizes anticodon nucleotides at positions 34 (C or U at a wobble position) and 35 (U) but not at position 36 (G for tRNA^Gln^ and C for tRNA^Glu^). It therefore seems possible that, in *T. tripolitanus* mitochondria, tRNAs expressed from both the tRNA^Gln^_1_ and tRNA^Gln^_2_ genes could be charged with glutamic acid by the mitochondrial non-discriminating GluRS.

Recently, the crystal structure of the bacterial ‘glutamine transamidosome complex’, consisting of tRNA^Gln^, GluRS, and AdT, indicated that glutamylation and transamidation may be consecutive reactions and that GluRS and AdT may take on conformational changes to compete for the acceptor stem of tRNA^Gln^ as their reaction target [38]. The same study showed that the bacterial AdT does not interact with the anticodon nucleotides of its substrate tRNA^Gln^ but recognizes the tRNA^Gln^-specific tertiary structure at an outer corner of the L-shaped tRNA^Gln^, especially in the D loop side.

**Figure 6:**
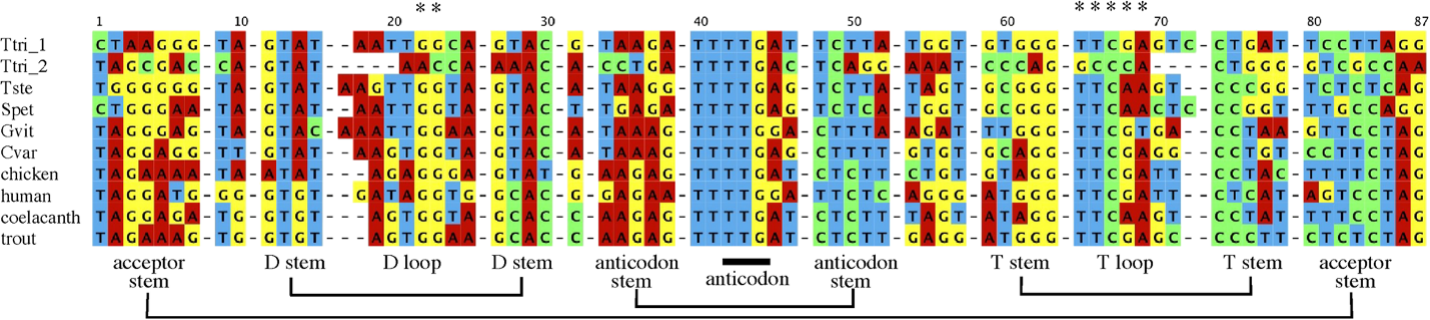
**Mitochondrial tRNA** ^**Gln**^ **gene sequences for geckos and other vertebrates.** tRNA^Gln^ gene sequences are aligned based on the standard clover-leaf structures. Asterisks indicate positions corresponding to conserved nucleotides for G_18_G_19_ in the D loop and T_55_T_56_C_57_R_58_A_59_ in the T loop (see text). Abbreviations and data sources are: tRNA^Gln^_1_ (Ttri_1; this study) and tRNA^Gln^_2_ (Ttri_2; this study) genes of *Tropiocolotes tripolitanus*, *Tropiocolotes steudneri* (Tste; this study), *Stenodactylus petrii* (Spet; this study), *Gekko vittatus* (Gvit; accession No. AB178897), *Coleonyx variegatus* (Cvar; AB114446), chicken (X52392), human (J01415), coelacanth (U82228), and trout (L29771).

It is well known that nonmitochondrial tRNAs conserve several nucleotides that are involved in forming the rigid L-shaped structure by tertiary hydrogen bondings, such as G_18_-Ψ_56_ (T_56_ at the DNA level) and G_19_-C_57_. Sequence comparison of mitochondrial tRNA^Gln^ genes from various vertebrates indicated that G_18_G_19_ in the D loop and T_55_T_56_C_57_R_58_A_59_ in the T loop are well conserved (Figure 6) while many other mitochondrial tRNA genes do not conserve these bases [22]. This observation is consistent with a view that formation of a standard L-shaped tertiary structure is necessary for a tRNA^Gln^ to be catalyzed by the mitochondrial AdT. *Tropiocolotes tripolitanus* tRNA^Gln^_1_ conserves these bases for the tertiary interactions but tRNA^Gln^_2_ does not (Figure 6). The latter even truncates nucleotides in the D and T loops considerably. Because mitochondrial tRNAs lacking the D loop/T loop interactions take on severely loosened tertiary structures [39, 40], they may not be a good substrate for the transamidation reaction catalyzed by the mitochondrial AdT.

Taken together, these results suggest that both tRNA^Gln^_1_ and tRNA^Gln^_2_ are possibly glutamylated in *T. tripolitanus* mitochondria but that only Glu-tRNA^Gln^_1_ may be efficiently converted to Gln-tRNA^Gln^_1_ for the protein synthesis. This implies that Glu-tRNA^Gln^_2_ possibly remains as an inactive form or has become a harmful reagent that can decode CAR codons as glutamic acid, rather than glutamine. In this regard, Nagao et al. [41] found that human mitochondria do not allow Glu-tRNA^Gln^ to participate in protein synthesis because it is not efficiently recognized by mitochondrial elongation factor Tu. Thus, Glu-tRNA^Gln^_2_ might simply be a harmless byproduct in *T. tripolitanus* mitochondria. Alternatively, though less likely, the mitochondrial AdTs may be able to recognize both tRNA^Gln^_1_ and tRNA^Gln^_2_ by a different mechanism from that of the bacterial AdTs. Metazoan mitochondrial aminoacyl-tRNA synthetases were suggested to have simplified recognition mechanisms towards substrate tRNAs in response to the decrease of structural constraints on mitochondrial tRNAs [39]. We therefore cannot rule out the possibility that the tRNA^Gln^_2_ gene serves as the second functional tRNA^Gln^ gene in *T. tripolitanus* mitochondria.

### Source of tRNA^Glu^for protein synthesis in *U. ebenaui*mitochondria

We found that the *Uroplatus ebenaui* mitogenome apparently lacks the tRNA^Glu^ gene. If so, how could protein synthesis be performed in *U. ebenaui* mitochondria? GAA and GAG are two codons for glutamic acid in the vertebrate mitochondrial genetic code [4] and no genetic code change is suggested for these codons in *U. ebenaui* mitochondria (Additional file 4: Table S3). Because glutamic acid codons (GAA + GAG) appear in protein-coding genes of the *U. ebenaui* mitogenome as frequently as in those of the mitogenomes of 17 geckos (Table 2), there must be a tRNA^Glu^ that is responsible for decoding these codons.

The most straightforward explanation is an import of nuclear-encoded cytosolic tRNA^Glu^ into the mitochondrion. The import of cytosolic tRNAs into vertebrate mitochondria has been suspected for some taxa. For example, marsupial mitogenomes do not appear to encode functional tRNA^Lys^ genes [42]. Biochemical experiments later supported the import of cytosolic tRNA^Lys^ into marsupial mitochondria [43]. The import of cytosolic tRNAs into mitochondria is more common in non-vertebrates (reviewed in [44]). The majority of tRNAs necessary for mitochondrial protein synthesis are not encoded by the *Tetrahymena* mitogenome and are imported from the cytosol [45, 46].

It seems noteworthy in this regard that the relative frequency of GAA vs. GAG codons in the *U. ebenaui* mitogenome is very strongly deviated (*p* < 0.001) from that averaged among 17 geckos, whereas there is no significant (*p* < 0.05) deviation of this relative frequency for *U. fimbriatus* and two *Tropiocolotes* species (Table 2). In general, codon usage in an organism reflects various factors, such as GC content of the genome and the relative abundance and translational efficiency of tRNAs that decode different codons (e.g., [47, 48]). In animal mitochondria, strand-specific base composition bias was also proposed as a major factor [49]. If *U. ebenaui* mitochondria do use tRNA^Glu^(s) imported from the cytosol, it seems possible that the usage of GAA and GAG codons is adapted to the different codon-decoding abilities of the imported cytosolic tRNA^Glu^(s) with regard to GAA and GAG.

An alternative explanation is that post-transcriptional modifications may create a tRNA^Glu^ from other tRNA genes. Enzymatic modification of anticodon bases of tRNAs could change decoding specificity from one amino acid to another (reviewed in [50]). RNA editing could also change the decoding specificity of tRNAs. RNA editing is known to occur in various metazoan mitochondrial tRNAs ([51, 52] and refs. therein). In marsupial mitochondria, a C to U editing at the second anticodon position switches a tRNA^Gly^ (anticodon GCC) to tRNA^Asp^ (anticodon GUC) [53]. Similar RNA editing of a G to C change at the third anticodon base of tRNA^Gln^ might supply the missing tRNA^Glu^ for *Uroplatus* mitochondria. However, this RNA editing would need to occur concomitantly with other modifications in, e.g., the D arm, in order not to be converted from Glu-tRNA^Glu^ to Gln-tRNA^Glu^ by transamidation (see the preceding section for the recognition sites by AdT).

## Conclusions

In the present study, seven new mitogenomic sequences were determined from Gekkonidae with the aid of high-throughput sequencing. Several new gene rearrangements were found and Gekkonidae can no longer be considered a group in which mitochondrial gene rearrangements rarely occur. Although the high-throughput sequencing has a weak point in assembling repeat sequences, we were able to demonstrate that moderately repetitive sequences, as found in the *T. tripolitanus* mitogenome, can be reliably assembled by this method. In future, high-throughput sequencing will further contribute to efficient and accurate mitogenomic sequencing from numerous metazoan taxa.

Although mitochondrial gene rearrangements have been described in various taxa, an intermediate type of gene arrangement that is indicative of molecular evolutionary mechanisms is rarely found. The unique gene arrangement found in the *T. tripolitanus* mitogenome provides an opportunity to study relatively new gene rearrangements in which the duplicate state of genes is maintained. Based on the characterization of duplicated genes (Additional file 2), the order of genes for tRNA^Arg^, ND4L, and ND4 (as in the typical gene organization) may be changed to ND4L, tRNA^Arg^, and ND4 after complete deletion of redundant genes or pseudogenes (Figure 5). However, if the duplicate pseudogenes retain a functional role in translating overlapping genes, they may not be deleted from the mitogenome, as seen in parrotfish mitochondrial tRNA pseudogenes that are retained as punctuation marks for mRNA processing [54].

In addition, the *T. tripolitanus* mitogenome may have gone through tRNA gene reassignment from tRNA^Arg^ to tRNA^Gln^ by a point mutation at the second anticodon position (Figure 5), although the novel tRNA^Gln^ gene may not be fully functional in translation. Mitochondrial tRNA gene reassignment has been reported in some invertebrates (e.g., [55]) but, to the best of our knowledge, it is uncommon in vertebrates. Together with the finding of tRNA^Glu^ gene loss in the *U. ebenaui* mitogenome, these new features should broaden our understanding of the evolution of mitochondrial gene arrangements.

## Methods

### Samples and general experimental procedures

*Lepidodactylus lugubris* sample was collected at Chichi-jima Island of the Bonin Islands, Japan. Other animal samples of either dead or live individuals were obtained from local shops or animal dealers in Japan. All experiments in which live animals are handled were conducted carefully under the guideline of the Animal Experiment Committee of Nagoya City University with permission (No. H21N-02).

A small amount of tissue from the tail muscle was used for crude DNA extraction with a DNeasy Tissue Kit (Qiagen). PCR amplifications were conducted with SpeedSTAR HS DNA polymerase (Takara) or PrimeSTAR GXL DNA polymerase (Takara) according to the manufacturer’s instructions. The former was routinely used for <2 kbp amplifications and the latter was selected for longer amplifications. Short amplified products were purified with a High Pure PCR Cleanup Micro Kit (Roche), followed by the dye termination sequencing reaction using a BigDye Terminator v3.1 Cycle Sequencing Kit (Life Technologies). The resultant reaction mixture was ethanol precipitated and applied to the 8-capillary 3500 Genetic Analyzer (Life Technologies) in the standard run mode.

### Complete mitochondrial genome sequencing

Crude extracted DNA from a tiny amount of animal tissue was used as template for long PCR amplification that nearly covered an entire mitochondrial genome (see Additional file 1: Table S1 for primer sequences used for the long PCR amplifications for each taxon). Approximately 2 μg of amplified products were pooled and sonicated using a Bioruptor UCD-250 (Cosmo Bio) into shorter fragments (~600 bp on average). After exclusion of short DNAs (<200 bp) by binding to Solid-Phase Reversible Immobilization beads (Agencourt AMPure XP: Beckman Coulter) [56, 57], recovered DNAs were end-repaired with T4 polynucleotide kinase (Takara) and T4 DNA polymerase (Takara) using the manufacturer’s protocol.

Using the parallel tagged sequencing method described by Meyer et al. [57], palindromic 20-bp DNAs, whose sequences differ from species to species, were ligated to both 5’ and 3’ ends of the repaired products. Ligated products from multiple species were quantified simultaneously with a Quant-iT Picogreen dsDNA Assay Kit and the Qubit Fluorometer (Life Technologies) to combine them at a nearly equal molar ratio. The pooled DNAs were digested with an 8-bp recognizing restriction enzyme, SrfI (Stratagene), at the central position of the ligated tag sequences. The final product (>500 ng) was sent to Hokkaido System Science Co. for Roche GS FLX Titanium high-throughput DNA sequencing.

Roche GS FLX Titanium sequencing produced short reads of 300–400 bp in length, which were then sorted into individual species based on the attached index tag sequences. After tags were removed, reads were assembled by the GS De Novo Assembler (Roche) into one to several contigs. Highly repetitive sequences inside the control region of mitochondrial genomes are not usually assembled together. In addition, short regions between the two long PCR primers (see above) are absent. These gap regions were amplified with reptile-oriented primers [58] or species-specific primers (data not shown), sequenced with the 8-capillary 3500 Genetic Analyzer (Life Technologies), and finally assembled into a contiguous circular mitogenome sequence with Sequencher 4.8 (Gene Codes).

The pyrosequencing chemistry adopted by Roche GS FLX Titanium sequencing has a weak point with homopolymer sequences (repeats of a single base) that tend to be miscalled. Majority-rule consensus sequence was trusted only when each site is covered by many reads (typically >20 reads) and ambiguous regions, if any, were independently amplified and sequenced by the Sanger method to confirm their sequences. Note that the accuracy of the above-mentioned method for sequencing a mitogenome was confirmed using several animal species in which their complete mitogenomic sequences for the same individuals had been determined in our laboratory by Sanger sequencing alone (Kumazawa, Y., unpublished data). The determined mitogenomic sequences (Table 1) did not include any unexpected frameshifts or stop codons inside protein-coding genes, except for the potential pseudogenes of *T. tripolitanus*.

### Analysis of gene arrangement and codon usage

Genes encoded in the determined mitogenomic sequences were identified by initial characterization with DOGMA [28] and subsequent manual inspections of gene structure, especially in light of secondary structures for mitochondrial tRNA genes [22]. In addition, we used the software Getmitogenome [59] to excise nucleotide sequences of 37 encoded genes, as well as amino acid sequences of 13 protein genes, which were added to a pre-existing alignment dataset for >150 vertebrates (data not shown). This procedure helped us to evaluate gene boundaries more carefully and to identify possible pseudogenes. Tandem repeats and CSB motifs [21] in the control region were identified with DNASIS-Mac ver. 3.5 (Hitachi Software Engineering).

Codon usage at codons for glutamine and glutamic acid was calculated using MEGA 5 [60]. Statistical significance was evaluated using the chi-square test with 5% significance level. First, the relative frequency of glutamine codons (CAA + CAG) vs. glutamic acid codons (GAA + GAG) was assumed to be equal from species to species as a null hypothesis. Deviation of the relative frequency in a species from that averaged among 17 gecko species was evaluated with the chi-square test. Second, the relative frequency of CAA vs. CAG codons was assumed to be equal from species to species as a null hypothesis and deviation of the relative frequency in a species from that averaged among 17 gecko species was evaluated in the same way. Finally, the same test was conducted to evaluate deviation of the relative frequency of GAA vs. GAG codons.

### Availability of supporting data

All nucleotide sequences and annotations reported in this work will be publicly available in the DDBJ nucleotide sequence database with accession numbers shown in Table 1 and Additional file 4: Table S2.

## Electronic supplementary material

Additional file 1: Table S1: Primers used in this study. **Figure S1.** Location of primers used to verify the gene arrangement of *T. tripolitanus* mitogenome. **Figure S6.** Four tRNA^Leu^(UUR) genes found in the *S. petrii* mitogenome. (PDF 356 KB)

Additional file 2: Figure S2: Alignment of ND4L gene sequences of geckos. **Figure S3.** Alignment of ND4L amino acid sequences of geckos. **Figure S4.** Alignment of ND4 gene sequences of geckos. **Figure S5.** Alignment of ND4 amino acid sequences of geckos. (PDF 6 MB)

Additional file 3: Figure S7: 1% agarose gel electrophoresis of PCR products amplified from closely related gecko species using rND2-4L and H5934m. **Figure S8.** 1% agarose gel electrophoresis of PCR products amplified from closely related gecko species using rND3-1L and rCUN-3H. (PDF 141 KB)

Additional file 4: Table S2: Sequences determined from closely related species in this study. **Table S3.** Evidence for no genetic code change in glutamine and glutamic acid codons for four gecko species. (PDF 83 KB)

## Abbreviations

AdT: Amidotransferase
AT6 and AT8: ATPase subunit 6 and 8
CO1-3: Cytochrome oxidase subunit 1-3
CSB: Conserved sequence block
cytb: Cytochrome *b*
GlnRS: Glutaminyl-tRNA synthetase
GluRS: Glutamyl-tRNA synthetase
mitogenome: Mitochondrial genome
ND1-6: NADH dehydrogenase subunit 1-6
O_L_: Light-strand replication origin
TDRL: Tandem duplication-random loss.
