# Supplementary material for: Gene rearrangements in gekkonid mitochondrial genomes with shuffling, loss, and reassignment of tRNA genes
Source: BMC Genomics. 2014 Oct 24;15(1):930. doi: 10.1186/1471-2164-15-930 (PMC4223735; doi:10.1186/1471-2164-15-930)
Supplement: Supplementary file 1 — Additional file 1: Table S1: Primers used in this study. Figure S1. Location of primers used to verify the gene arrangement of T. tripolitanus mitogenome. Figure S6. Four tRNALeu(UUR) genes found in the S. petrii mitogenome. (PDF 356 KB) [file 12864_2014_6628_MOESM1_ESM.pdf]

Table S1. Primers used in this study

name	length	sequence (5' to 3')
L12S-3L	28	GTAAACTTCGTGCCAGCCACCGCGGTTA
L16S-2L	29	ATTAAYAGTCCTACGTGATCTGAGTTCAG
L16S-4L	26	CGGAGYAATCCAGGTCGGTTTCTATC
r16S-4L	20	TACTCCAGGGATAACAGCGC
rMet-1L	20	TAAGCTWTYGGGCCCATACC
L12S-4H	27	TCCAGTACRCTTACCATGTTACGACTT
L16S-1H	29	GACAARTGATTAYGCTACCTTTGCACGGT
L16S-1Ht	29	GACAAGTGATTACGCTACCTTTGCACGGT
r16S-2H	19	GATRGCGCTGTTATCCCTG
cytb2-3	29	GTTGCCCCTCAGAAKGATATTTGTCCTCA
rND3-1L	19	ATGAATGCGGMTTTGAYCC
rCUN-3H	20	CTTTCACYTGGARTTGCACC
rND2-4L	22	TAACAGGMTTYATRCCAAAATG
H5934m	34	CCCGACGCTGCAGGGTGCCAATGTCTTTRTGRTT
uND5-2L	23	GAACAAGACATYCGAAAAATAGG
rND6-3L	20	GCAACWGAATAHGCAAATAC
ucytb-1H	26	GCCCCTCAGAATGATATTTGTCCTCA
uThr-2L	21	AAAGCGYTGGTCTTGTAAACC
rPhe-3H	20	GCAACTTCAGGGCCGTGCTT
r12S-1H	21	TRTAACCGCGGTKGCTGGCAC
Ttri-4L	18	GAAATCACACTACTGCTG
Ttri-5L	17	CACCACGCAGCCTCATT
Ttri-6L	18	ATTTTACCACTTTTAGGC
Ttri-7L	16	GGATCAATAGTCCTTG
Ttri-8L	18	CACACAAACTCTCGCCAT
Ttri-4H	17	GAATTAGGGTGGTCTCA
Ttri-5H	17	TAGCAGGTTTAGGGCCT
Ttri-6H	17	TTGCTCCTCAAAGGGTC
Ttri-7H	16	TTTTATGTCCACAGCG
Ttri-8H	17	CTGCTCCTCAGAGGGCT

Combination of primers for long PCR amplification of mitogenome was: L16S-4L and L16S-1Ht for *T. tripolitanus*, r16S-4L and L12S-4H for *T. steudneri*, rMet-1L and r16S-2H for *S. petrii*, L16S-4L and L12S-4H for *L. lugubris*, L16S-2L and L16S-1H for *P. guimbeaui* and *U. fimbriatus*, and L12S-3L and cytb2-3 for *U. ebenaui*.

r16S-4L, rND6-3L ucytb-1H, and r12S-1H primers were first described by Kumazawa and Endo (2004) DNA Res. 11: 115-125.

rMet-1L primer was first described by Amer et al. (2012) Comp. Funct. Genom. 2012:851379.

H5934m was modified from H5934 reported in Macey et al. (1997) Mol. Biol. Evol. 14: 91-104.

uND5-2L was modified from rND5-2L reported in Kumazawa and Endo (2004) DNA Res. 11: 115-125.

rPhe-3H was modified from rPhe-2H reported in Amer and Kumazawa (2005) Gene 346: 249-256.

uThr-2L was modified from uThr-1L reported in Dong and Kumazawa (2005) J. Mol. Evol. 61: 12-22.

All other primers were newly designed for this study.

Primers starting with 'Ttri' are specific for *Tropiocolotes tripolitanus* (see Fig. S1 for locations).

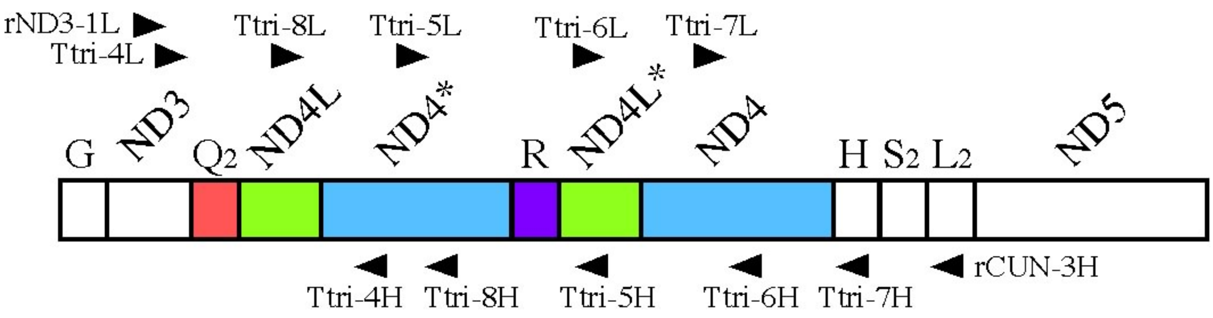

Fig. S1. Location of primers used to verify the gene arrangement of *T. tripolitanus* mitogenome

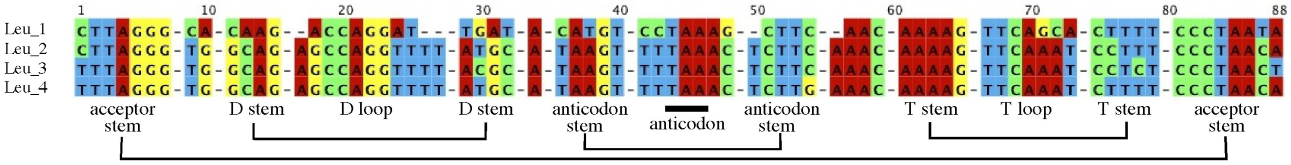

Fig. S6. Four tRNA^{Leu}(UUR) genes found in the *S. petrii* mitogenome. Four tandemly duplicated genes are numbered from Leu_1 to Leu_4.
