# Supplementary material for: Gene rearrangements in gekkonid mitochondrial genomes with shuffling, loss, and reassignment of tRNA genes
Source: BMC Genomics. 2014 Oct 24;15(1):930. doi: 10.1186/1471-2164-15-930 (PMC4223735; doi:10.1186/1471-2164-15-930)
Supplement: Supplementary file 2 — Additional file 2: Figure S2: Alignment of ND4L gene sequences of geckos. Figure S3. Alignment of ND4L amino acid sequences of geckos. Figure S4. Alignment of ND4 gene sequences of geckos. Figure S5. Alignment of ND4 amino acid sequences of geckos. (PDF 6 MB) [file 12864_2014_6628_MOESM2_ESM.pdf]

```
                            10         20         30         40         50
Ttri_1      1 ---ATGCCCT TAATACAATT CACAGCCTAC GCTACCTTTT TCACTGGACT    50
Ttri_2      1 ATAATGCCCT CAA------- CGCAACCTTC ACCACCTTTT TCTCTGGGCC    50
Tste        1 ---GTGACCA TCGCTCAATT TTTAATATAC ACCACCTTTA CGCTAGCCAT    50
Gvit        1 ---ATGATAC TTACCCACTT TGCGGTAACT GCTACATTCA TACTAAGTAC    50
Cvar        1 ---ATGACAA ACATGCAATT CACCATAACA CTAGCCTTTA TCCTCAGCAT    50

                            60         70         80         90        100
Ttri_1     51 AACTGGACTA ATTCTATACC GTAAACATCT TATCTCCGCC CTACTATGCC   100
Ttri_2     51 AGCCAAACCA GCTCTATGCC GAAAGCATCC TATCTCCACC CTACTATGCC   100
Tste       51 AGCGGGACTA ATCCTATACC GCAAACATCT AATTTCCGCC CTGCTATGCC   100
Gvit       51 CTTGGGCCTA GCCTTTCACC GAAAACATCT TGTATCTGCC TTACTTTGCA   100
Cvar       51 TTCAGGGCTA GCAATACACC GCACGCATCT TGTATCTGCC CTCCTATGCA   100

                           110        120        130        140        150
Ttri_1    101 TAGAAAGCCT TATGGTTGCT CTATTTATCA CCTTTACCCT C-------CA   150
Ttri_2    101 CATAAAGCCC CATTGTTGCT CTATTTATTA CCCTGACCCC CATATATTTT   150
Tste      101 TAGAAGGACT TATAATTACC CTGTTCTTCG CCCTCACAAC C-------AT   150
Gvit      101 TTGAGGGTAT AATGTTAGCA CTATTTTTAG CGCTAACAGT T-------AA   150
Cvar      101 TTGAAACTAT AATACTGGCC CTATTTATAT TCCTAGTCTT A-------CT   150

                           160        170        180        190        200
Ttri_1    151 CACACAAACT CTCGCCATCA CATCAACAGC ACTACAACCC GTAATTATAC   200
Ttri_2    151 ACCACTTTTA GGCCCTAAAC CTGCTAACAT GCTAAAAACC CTAATCACAG   200
Tste      151 CTCACAAAGC CTATTTACTA CTACAACCAC AATTCAACCA ATCATCCTAC   200
Gvit      151 TTCTCAAACA CTTAGTCTAA CTACAATATC TCTACATCCT ATCACTTTAC   200
Cvar      151 AGCACAAAAC ACAGAACTAA CAACCAACGC CCTATTTCCA ATAGCCCTTC   200

                           210        220        230        240        250
Ttri_1    201 TAACACTCTC AGCATGCGAG GCTGGAACTG GGCTAGCCCT CCTAGTTGCA   250
Ttri_2    201 CCGCGTTGCT GTTTTTTTTT GTCCTGGCTC TGCCACAAAA AAACAGTCAG   250
Tste      201 TTACACTTTC AGCATGTGAA GCAGGGACAG GACTGGCCCT ACTAGTAGCA   250
Gvit      201 TCACGTTGGC AGCCTGTGAA GCAGGATCGG GCCTAGCCTT ACTTGTTGCT   250
Cvar      201 TAACCTTCTC GGCTTGCGAG GCGAGCACAG GGCTTGCCAT TCTAGTTGCA   250

                           260        270        280        290        300
Ttri_1    251 TCATCCCGCA CCCATGCATC TGACCACTTA AAGGCCCTAA ACCTGCTAAT   300
Ttri_2    251 CGCTGCTGTT TTTTTTTGTC CTGGCTCTGC CACACAAAAT TTTATCCCCA   300
Tste      251 TCCTCACGCA CCCATGCCTC CGACCACCTA AAAACCATAA ATTTATTGAT   300
Gvit      251 TCTTCCCGAA CTCACGCCTC TGATAATCTA AAAGCCCTTA ATCTCCTAAA   300
Cvar      251 TCAACACGAA CCCACGCCTC AGACCACCTA AAAACCATAA ACTTATTACA   300

                           310        320        330        340        350
Ttri_1    301 ATGC...... .......... .......... .......... ..........   350
Ttri_2    301 ACAG...... .......... .......... .......... ..........   350
Tste      301 ATGT...... .......... .......... .......... ..........   350
Gvit      301 ATGT...... .......... .......... .......... ..........   350
Cvar      301 ATGT...... .......... .......... .......... ..........   350
```

Fig. S2. Alignment of ND4L gene sequences of geckos. *Tropiocolotes tripolitanus* ND4L gene next to the tRNA$^{Gln}_{2}$ gene (Ttri_1; this study), *Tropiocolotes tripolitanus* ND4L pseudogene next to the tRNA^{Arg} gene (Ttri_2; this study), as well as ND4L genes from *Tropiocolotes steudneri* (Tste; this study), *Gekko vittatus* (Gvit; accession No. AB178897) and *Coleonyx variegatus* (Cvar; AB114446) are compared and aligned.

```
                      10         20         30         40         50
Ttri_1       1 -MPLMQFTAY ATFFTGLTGL ILYRKHLISA LLCLESLMVA LFITFTLHTQ    50
Ttri_2       1 MMPSTQPSPP FSLGQPNQLY AESILSPPYY AHKAPLLLYL LPWPPYILPL    50
Tste         1 -MTIAQFLMY TTFTLAMAGL ILYRKHLISA LLCLEGLMIT LFFALTTISQ    50
Gvit         1 -MMLTHFAVT ATFMLSTLGL AFHRKHLVSA LLCIEGMMLA LFLALTVNSQ    50
Cvar         1 -MTNMQFTMT LAFILSISGL AMHRTHLVSA LLCIETMMLA LFMFLVLLAQ    50

                      60         70         80         90        100
Ttri_1      51 TLAITSTALQ PVIMLTLSAC EAGTGLALLV ASSRTHASDH LKALNLLMC.   100
Ttri_2      51 LGPKPANMLK TLITAALLFF FVLALPQKNS QRCCFFLSWL CHTKFYPQQ.   100
Tste        51 SLFTTTTTIQ PIILLTLSAC EAGTGLALLV ASSRTHASDH LKTMNLLMC.   100
Gvit        51 TLSLTTMSLH PITLLTLAAC EAGSGLALLV ASSRTHASDN LKALNLLKC.   100
Cvar        51 NTELTTNALF PMALLTFSAC EASTGLAILV ASTRTHASDH LKTMNLLQC.   100
```

Fig. S3. Alignment of ND4L amino acid sequences of geckos. Amino acid translation of ND4L gene sequences from Fig. S2 are compared and aligned. Indels are removed for the in-frame translation of Ttri_2. For taxon abbreviations, see the legend of Fig. S2.

```
                                  10         20         30         40         50
Ttri_1       1  ATGCTAAAAA CCCTAATCCC AACAGCGCTG CTGGTTTTTT CCGTCCTGGC    50
Ttri_2       1  ATGCTAAAAA CCCTAATCCC AACAGCGCTG CTGTTTTTTT CCGTCCTGGC    50
Tste         1  ATGTTAAAAG CCCTCGTCCC AACAGCACTA CTTTTCCCTA CTATCCTGGC    50
Gvit         1  ATGTTAACAC TATTATTCGC AACTAGCATA ATTATTCCCA CAGCTTTATT    50
Cvar         1  ATGTTAAAAA TCCTACTACC AACAGTCATA CTAGCCCCAA CAACAATATT    50

                                  60         70         80         90        100
Ttri_1      51  TCTGCCACAC AAAATCTTAT TCCCAACAGT AACTGCATAC TCAATCCTAA   100
Ttri_2      51  TCTGCCACAC AAAATTTTAT TCCCAACAGT AACTGCATAC TCAATCCTAA   100
Tste        51  TCTGCCACAC AAACTTCTTT ACCCAGCAGC TACTGCGTAC TCTACGCTAA   100
Gvit        51  AATAAATACT AAACACCTAT TTACCACAAC CACTACTCAC TCTATAATTA   100
Cvar        51  AATCCCCAGC CCCCTCCTAT TTTTAACCTT CACAGGCTAC TCAATACTCA   100

                                 110        120        130        140        150
Ttri_1     101  TCGCCATAAC CACCCTAAAA TGGCTGTACT CCCCCATGAA CATGAACCCA   150
Ttri_2     101  TCGCCATAAC CACCCTAAAA TGGCTATACT CCCCCATGAA CATGAACCCA   150
Tste       101  TCTCCGCCTA CACACTAAAA TTACTATATT CACCCCTCAA CCTAAAACCA   150
Gvit       101  TTACGATATT TATGCTTAAA TGATTATACC AACCACTTAA CCAAAAACCT   150
Cvar       101  TTGCACTATT CAGCACTAAC TATCTAAACG CACCGCTCAA CCCAAAACTA   150

                                 160        170        180        190        200
Ttri_1     151  TACATGATCA ACCAATGAAT GATCACAGAC GAAATCTCAA CCCCACTCAT   200
Ttri_2     151  TACATGATCA ACCAATGAAT GGTCACAGAC GAAATCTCAA CTCCACTCAT   200
Tste       151  CAAACCATCT CCCCCCTCAC AATAACAGAC AAAATCTCAA CCCCACTTCT   200
Gvit       151  TATAATATTA ATACTCTTAT AGCATTAGAT AATATTTCCA CCCCTTTAAC   200
Cvar       151  CATTTCACCA ACACCATCAT AGGCCTAGAC CACATCTCAA CCCCCCTACT   200

                                 210        220        230        240        250
Ttri_1     201  TATCATATCT GCTTGAATCC TCCCACTAAC AACAATCGCC AGTCAAAACA   250
Ttri_2     201  TATCCTATCT GCTTGAATCC TCCCACTAAT AACAATCGCC AGTCAAAATA   250
Tste       201  AATCCTGTCA TCCTGACTGC TCCCAATAAT AATCATTGCC AGCCAACATG   250
Gvit       201  CATCATGTCC GCTTGAATTC TGCCCCTAAT AATCGTTGCA AGTCAACACC   250
Cvar       201  AATCCTTTCA GCCTGGCTTC TACCACTCAT AGCAATTGCA AGCCAATACC   250

                                 260        270        280        290        300
Ttri_1     251  GCCTTTCCAC AGAACCCCTG CCACGTAAAC GCATATTCCT TGCCGCAGCA   300
Ttri_2     251  GCCTTTCCAC AGAACCCCTG CCACGTAAAC GCATATTCCT TGCCGCAGCA   300
Tste       251  CCCTAAACCA CGAACCAATA ATCCGAAAAC GAACATTTAT TGCTACCGCA   300
Gvit       251  ATCTAATTAA CGAACCTATT AATCGAAAAC GATTATTTAT TACTACATGT   300
Cvar       251  ATCTTAAAAA TCAACCAGCC ACTCGCAAAC GAACATTCTT AATAACCACC   300

                                 310        320        330        340        350
Ttri_1     301  ACCACCCTAC AAACTACCCT AATTTTAGCC CTTACCGCAC CAGACATAAC   350
Ttri_2     301  ACCGCCCTAC AAACTACCCT AATCTTAGCC TTTACCGCCC CAGACATAAC   350
Tste       301  GCAATACTAC AAACAGCCCT AATAATAACT TTCATTGCAC CAGATATAAC   350
Gvit       301  GCAATTCTAC AGACAACTTT AATCTTAACC TTTACCACAA CAAATCTACT   350
Cvar       301  GCACTACTTC AATCCACACT AGTCTTAACA TTCTCAGCAA CTGATCTTAT   350

                                 360        370        380        390        400
Ttri_1     351  AATATTTTAC ATTATATTTG AGACCACCCT AATTCCAACT CTCCTCCTAA   400
Ttri_2     351  AATATTTTAC ATCATATTCG AGGCCACCTT AATCCCAACT CTCCTTCTAA   400
Tste       351  AATATTCTAC ATCATATTTG AAGCAACCCT AGTACCAACC CTATTCATAA   400
Gvit       351  CCTTTTCTAC ATTATATTTG AAGCTACTTT AATCCCAACT TTGATCTTAA   400
Cvar       351  AACATTCTAC ATTATATTCG AGGCAACACT AATCCCCACC CTTATCCTCA   400

                                 410        420        430        440        450
Ttri_1     401  TCACTCGATG AGGCACCCAA ACAGAGCGAC TGGGTGCCGG AATACACTTC   450
Ttri_2     401  TCACTCGATG AGGCACCCAA ACAGAGCGAC TAGGTGCCGG AATATACTTC   450
Tste       401  TCACCCGCTG AGGGGCACAA GCAGAGCGAC TAAACGCAGG AACATACTTT   450
Gvit       401  TCACACGCTG AGGTAGTCAA CCAGAACGGT TAAATGCTGG AACATATTTC   450
Cvar       401  TCACCCGATG AGGAAATCAG GCCGAACGAC TAAATGCAGG CACATACTTT   450

                                 460        470        480        490        500
Ttri_1     451  TTATTTTATA CACTAGCCGG ATCACTACCA CTGTTAATTG TTATCCTCAA   500
Ttri_2     451  TTATTTTATA CACTAGCCGG ATCACTACCA CTGTTAATTG TTATCCTCAA   500
Tste       451  CTATTTTACA CCCTGACTGG ATCCCTTCCA CTACTAATTG CTATTCTAAC   500
Gvit       451  TTATTTTATA CCCTAATTGG ATCTCTACCA TTATTAATTG CAATTTTGTT   500
Cvar       451  CTTTTCTACA CACTAGCAGG CTCCCTCCCA CTATTAATTG CAATCCTCCA   500
```

Fig. S4. Alignment of ND4 gene sequences of geckos. *Tropiocolotes tripolitanus* ND4 genes preceding the tRNA^{Arg} gene (Ttri_1; this study) and tRNA^{His} gene (Ttri_2; this study), as well as ND4 genes from *Tropiocolotes steudneri* (Tste; this study), *Gekko vittatus* (Gvit; accession No. AB178897) and *Coleonyx variegatus* (Cvar; AB114446) are compared and aligned.

```
                    510        520        530        540        550
Ttri_1      501  CACCCTAGAA AAAAACAACC ACACTGTCTT CATATTACTG GCCATGAAAC   550
Ttri_2      501  CACCCTAGAA AAAAACAACC ACACTGTCTT CATACTACTG GCCATAAAAC   550
Tste        501  ACTCTTATCA AAAACTAATC ACACCAATTT TGTACTACTA ACAATAAAAC   550
Gvit        501  TATATATAAC ACTAATAACC ACACTCTAAT ATTTACAACT GCACAAATAA   550
Cvar        501  TATTTACTCA ACCACCAACC TAACAACCAT GTTCATAATG GCAACTAACC   550

                    560        570        580        590        600
Ttri_1      551  AACAACCAAC CGTCACTCAC CACACCCATA TCATTATCTG ACTTGGATTC   600
Ttri_2      551  AACAACCAGC CATCACTCAC CACACCCATA TCATTATCTG ACTTGGATTC   600
Tste        551  AACAACCACT AGAGCCCTCT TACACCAACA ACATAATATG ACTAGCATGC   600
Gvit        551  CCCCT----- -GAAAATTTA AACATAACTA CAATTATATG ACTTGCTTGC   600
Cvar        551  AACAC----- -CCCCCATCA TGAACCACCA CAATAATATG GTTGGGCTGC   600

                    610        620        630        640        650
Ttri_1      601  ATATTCGCCC TATTAGTAAA AATACCACTC TACGGAGCCC ACCTATGACT   650
Ttri_2      601  ATATTCGCCC TATTAGTAAA AATACCACTC TACGGAGCCC ACCTATGACT   650
Tste        601  ATACTAGCCT TCCTAGTAAA AATACCACTA TACGGCACAC ACCTGTGATT   650
Gvit        601  ACATTAGCAT TTATAGTAAA AATACCACTA TATGGCCTAC ACCTGTGATT   650
Cvar        601  TTACTGGCCT TCTTAGTAAA AATACCCCTA TACGGCCTAC ACTTATGACT   650

                    660        670        680        690        700
Ttri_1      651  ACCAAAAGCC CACGTAGAAG CCCCAATTGC CGGATCAATA GTTCTTACCA   700
Ttri_2      651  ACCAAAAGCC CACGTAGAAG CCCCAATTGC CGGATCAATA GTCCTTGCCG   700
Tste        651  GCCAAAAGCA CATGTAGAAG CACCAATTGC AGGATCAATA ATCCTTGCCG   700
Gvit        651  ACCTAAAGCA CATGTAGAGG CACCCATCGC TGGATCAATA ATCCTAGCCG   700
Cvar        651  ACCAAAAGCC CATGTCGAAG CCCCAATTGC AGGTTCAATA GTCCTAGCCG   700

                    710        720        730        740        750
Ttri_1      701  CCGTACTACT AAAACTCGGA GGCTATGGAA TCATCCGCAT CATTTCAATC   750
Ttri_2      701  CCGTACTACT AAAACTCGGA GGCTATGGAA TCATCCGCAT CATTTCAATC   750
Tste        701  CCGTACTATT AAAGCTAGGA GGATATGGGA TTATTCGCCT TATGCCAACC   750
Gvit        701  CAGTCCTCCT AAAACTAGGC GGATATGGCA TCATTCGCAT AACACCAATA   750
Cvar        701  CAATCCTACT AAAGCTTGGT GGCTACGGCA TCATCCGCAT CACCCCAATC   750

                    760        770        780        790        800
Ttri_1      751  CTCCCGCCAA CACAAACACC ACGCAGCCTC ATTACAGCCC CCCTAACAAC   800
Ttri_2      751  CTCCCGCCAA CACAAACACC ACACAATATT ATCACAGCCC CCCTAACAAC   800
Tste        751  GTAACA---- ---------- ---------- ---AACATCA CACAAACATT   800
Gvit        751  TTACTA---- ---------- ---------- ---CACATAA CACAAACTAT   800
Cvar        751  CTACCC---- ---------- ---------- ---CCCCTAA CTCAAACAAT   800

                    810        820        830        840        850
Ttri_1      801  ACCCTACCCA TTCATCGTCC TAGCCCTCTG AGGAGCAGTC ATAACAAGCA   850
Ttri_2      801  ACACTACCCT TTTATCGTCC TGACCCTTTG AGGAGCAATC ATAACAAGCA   850
Tste        801  ATACCACCCA TTCATTGTAC TAGCCTTATG AGGAATAATT ATAACAAGCA   850
Gvit        801  ATACTTTCCA TTCATTGTAC TTAGTTTATG AGGAATAATT ATGACTAGCC   850
Cvar        801  CTACCTCCCA TTCATCATTC TTGCCCTCTG AGGAATAATT ATAACCAGCC   850

                    860        870        880        890        900
Ttri_1      851  TAATCTGCCT ACGCCAACCA GGCCTGAAAT CCATCATCAC CTACTCATCA   900
Ttri_2      851  TAATCTGCCT ACGCCAACCT GACCTAAAAT CCATCATCGC CTATTCATCA   900
Tste        851  TGATCTGCTT ACGACAAACA GACCTAAAAT CAATTATTGC TTACTCGTCA   900
Gvit        851  TAATTTGTCT ACGTCAGACA GATCTTAAAT CAATTATCGC ATACTCTTCC   900
Cvar        851  TAATCTGCCT GCGCCAAACA GACCTAAAAT CGATCATCGC ATATTCCTCC   900

                    910        920        930        940        950
Ttri_1      901  ATCAGCAACA CAGGCCTGGT TGTAGCCGCT GCCCTTATTC AAACGCCCTG   950
Ttri_2      901  ATTAGCCACA TAGGCCTGGT TGTAGCCGCT GCCCTTATTC AAACACCCTG   950
Tste        901  GTAAGCCACA TGGGCCTTGT TATCGCCGGC ACCCTAATTC AAACACCGTG   950
Gvit        901  GTTAGTCATA TAGGCTTAGT TATTGCAGCT ACTCTAATTA ATACACCATG   950
Cvar        901  GTAAGCCACA TAGGACTTGT AACTGCAGCT GCCCTCATCC ACACCCCATG   950

                    960        970        980        990       1000
Ttri_1      951  AAGCATCAAC AGCGCCATAC TACTAATAAT CACACACGAC CTTACATCCT  1000
Ttri_2      951  AAGCATTAAT GGCGCCATAT TACTAATAAT CGCACATGGC CTCACATCTT  1000
Tste        951  AGGCACAAAA GGAGCAATAA TCTTAATAAT TGCACATGGC CTCACATCTT  1000
Gvit        951  GAGTATTGCT GGGGCAATAA CTTTGATAAT TGCCCACGGA CTCACCTCAT  1000
Cvar        951  AAGTATCACA GGCGCAATAA TCTTAATAAT CGCACACGGC CTAACCTCTT  1000
```

Fig. S4. (continued)

```
                           1010       1020       1030       1040       1050
Ttri_1           1001 CTATACTTTT CTGCCTAGCA AACACTAACT ACGAGCGGAC CCACTCACGA   1050
Ttri_2           1001 CCATACTCTT CTGCCTAGCA AACACTAACT ACGAACGGAC CCACTCACGA   1050
Tste             1001 CAATACTATT CTGCCTAGCG AACACAAACT ACGAGCGCAC CCACACACGA   1050
Gvit             1001 CCGTACTTTT TTGCCTCGCT AACACAAATT ATGAGCGCAT GCACACACGA   1050
Cvar             1001 CAATACTTTT CTGCTTAGCA AACACCAACT ATGAGCGCAC CCACTCCCGA   1050

                           1060       1070       1080       1090       1100
Ttri_1           1051 ACACTAATCC TAACTCGAGG GATACACCTA GCACTACCCC TAATAGCCAC   1100
Ttri_2           1051 ACACTAGTCC TAACCCGAGG ACTACAACTA GCACTGCCCC TAATAACCAC   1100
Tste             1051 ACCCTAATCC TAACCCGAGG CCTTCAACTA GCACTCCCAC TAATAACCTC   1100
Gvit             1051 ACACTTTTAT TAACCCGAGG ATTACTATTC GCCTTACCAC TAATAACAAC   1100
Cvar             1051 ACACTACTAA TCGCCCGAGG CCTTCAACTA ATCCTACCAC TAATAACAAC   1100

                           1110       1120       1130       1140       1150
Ttri_1           1101 ATGATGAATA ATAGCAAGCA TAGCCAATAT GAGCCTACCC CCAACTATCA   1150
Ttri_2           1101 ATGATGACTA ACAGCAAGCC TAGCTAACAT GAGCCTGCCC CCAACTATCA   1150
Tste             1101 CTGATGAATA GCAGCTAGCC TTACAAACCT CGCCCTGCCC CCAACCATTA   1150
Gvit             1101 TTGATGATTA ATAGCAAGCC TTATAAATAT AGCCCTACCT CCAACAATCA   1150
Cvar             1101 TTGATGACTA ATGGCTAGTC TTGCCAACCT CGCCCTACCT CCAACAATCA   1150

                           1160       1170       1180       1190       1200
Ttri_1           1151 ATATGCTAGG AAAAATCACA ACTATCTCCT CTGCCTTCAG TTGAAACACA   1200
Ttri_2           1151 ATATGCTAGG AGAAATCATA ATTATCTCTT CTACCTTCAA TTGAAGCACA   1200
Tste             1151 ACCTGCTAGG AGAAATTATG ATTATCACCT CCATTTTTAA CTGAAACACA   1200
Gvit             1151 ATTTACTAGC AGAATTAATA ATTATTACTT CACTATTCAA TTGAAATACT   1200
Cvar             1151 ATCTAATAGG AGAGCTCATT ATTATCAGTA CCTTATTTAG CTGATCAACA   1200

                           1210       1220       1230       1240       1250
Ttri_1           1201 ACTACAAGTA TTTTAATAAC ACTAGCCACA CTACTTACCA CTGCATATTC   1250
Ttri_2           1201 ATAACAATTA TTTTAACAGC ACTAACCACA CTACTTACCG CTGCATATTC   1250
Tste             1201 ATTACCATCG CACTCACAGC CATAGCCACA GTACTTACAG CTACCTACTC   1250
Gvit             1201 TTAACAATTA TCTTAACCGG CACAACAACA CTAATCACCG CAACATATTC   1250
Cvar             1201 CCAACCATTC TCATCACAGG ACTAACCACC CTAATTACCG CTACATACTC   1250

                           1260       1270       1280       1290       1300
Ttri_1           1251 ACTACACTTC CTCATCACCC CCCTGCACAA CGAACTATCG ACACTAACCA   1300
Ttri_2           1251 ACTATACATC TTCAGCACCA CTCAACGCAG TAAACTATCA ACACCAACCA   1300
Tste             1251 CCTACACATC TTCCTAACCA CTCAACGCCA CAAACTATCA ACACGCACCC   1300
Gvit             1251 ATTATATATT TTTATATCAT CACAGTGAAG CACTTTACCA CCAAACAAAA   1300
Cvar             1251 CCTATATATC TTCCTGATAA CACAACGGAA TAAAATAATA AACCACCTAA   1300

                           1310       1320       1330       1340       1350
Ttri_1           1301 AAGTGCCCCC TACACAAACA CGAGAACATC TACTAATAAC CCTCCACCTC   1350
Ttri_2           1301 AACTACCCCC TACACAAACA CGCGAACACC TACTAATAAC CCTCCATCTC   1350
Tste             1301 CCCTAAAACC CACACACACA CGCGAACACC TATTAATAAC ACTCCACCTG   1350
Gvit             1301 CTCTATCAAT AACTCACACA CGAGAACACC TATTACTAAC TCTACATCTT   1350
Cvar             1301 CCTTCCCCCC CTCCCACACT CGAGAACACC TACTCATGTC CCTGCACATA   1350

                           1360       1370       1380       1390       1400
Ttri_1           1351 GCCCCCATAG CCGTATTTAC CTTCAGCCCA GACCTAACGC CACTCACATT   1400
Ttri_2           1351 ACCCCGATAA TCTTACTAAT CCTCAGCCCA AACCTAGTGC TC........   1400
Tste             1351 GCCCCACTAA TACTCCTAAT CCTACACCCA AACCTAATAC TA........   1400
Gvit             1351 ATCCCATTAA TAGCACTAAT TACTAATCCA AATTTAATAT TC........   1400
Cvar             1351 ATCCCCCTAA CATTACTTAT CCTCACCCCA AAACTCATAA TA........   1400

                           1410       1420       1430       1440       1450
Ttri_1           1401 TGATTTAATC GAGGAGTTCT ACCTATGAGC CCTCGGATTG GGC.......   1450
Ttri_2           1401 .......... .......... .......... .......... ..........   1450
Tste             1401 .......... .......... .......... .......... ..........   1450
Gvit             1401 .......... .......... .......... .......... ..........   1450
Cvar             1401 .......... .......... .......... .......... ..........   1450
```

Fig. S4. (continued)

```
                                 10         20         30         40         50
Ttri_1          1 MLKTLIPTAL LVFSVLALPH KILFPTVTAY SILIAMTTLK WLYSPMNMNP    50
Ttri_2          1 MLKTLIPTAL LFFSVLALPH KILFPTVTAY SILIAMTTLK WLYSPMNMNP    50
Tste            1 MLKALVPTAL LFPTILALPH KLLYPAATAY STLISAYTLK LLYSPLNLKP    50
Gvit            1 MLTLLFATSM IIPTALLMNT KHLFTTTTTH SMIITMFMLK WLYQPLNQKP    50
Cvar            1 MLKILLPTVM LAPTTMLIPS PLLFLTFTGY SMLIALFSTN YLNAPLNPKL    50

                                 60         70         80         90        100
Ttri_1         51 YMINQWMITD EISTPLIIMS AWILPLTTIA SQNSLSTEPL PRKRMFLAAA   100
Ttri_2         51 YMINQWMVTD EISTPLIILS AWILPLMTIA SQNSLSTEPL PRKRMFLAAA   100
Tste           51 QTISPLIMTD KISTPLLILS SWLLPMMIIA SQHALNHEPM IRKRTFIATA   100
Gvit           51 YNINTLMALD NISTPLTIMS AWILPLMIVA SQHHLINEPI NRKRLFITTC   100
Cvar           51 HFTNTIMGLD HISTPLLILS AWLLPLMAIA SQYHLKNQPA TRKRTFLMTT   100

                                110        120        130        140        150
Ttri_1        101 TTLQTTLILA LTAPDMTMFY IMFETTLIPT LLLITRWGTQ TERLGAGMHF   150
Ttri_2        101 TALQTTLILA FTAPDMTMFY IMFEATLIPT LLLITRWGTQ TERLGAGMYF   150
Tste          101 AMLQTALMMT FIAPDMTMFY IMFEATLVPT LFMITRWGAQ AERLNAGTYF   150
Gvit          101 ATLQTTLILT FTTTNLLLFY IMFEATLIPT LILITRWGSQ PERLNAGTYF   150
Cvar          101 ALLQSTLVLT FSATDLMTFY IMFEATLIPT LILITRWGNQ AERLNAGTYF   150

                                160        170        180        190        200
Ttri_1        151 LFYTLAGSLP LLIVILNTLE KNNHTVFMLL AMKQQPTVTH HTHIIIWLGF   200
Ttri_2        151 LFYTLAGSLP LLIVILNTLE KNNHTVFMLL AMKQQPAITH HTHIIIWLGF   200
Tste          151 LFYTLTGSLP LLIAILTLLS KTNHTNFVLL TMKQQPLEPS YTNNMMWLAC   200
Gvit          151 LFYTLIGSLP LLIAILFMYN TNNHTLMFTT AQMTP--ENL NMTTIMWLAC   200
Cvar          151 LFYTLAGSLP LLIAILHIYS TTNLTIMFMM AINQH--PPS WTTTMMWLGC   200

                                210        220        230        240        250
Ttri_1        201 MFALLVKMPL YGAHLWLPKA HVEAPIAGSM VLTTVLLKLG GYGIIRIISI   250
Ttri_2        201 MFALLVKMPL YGAHLWLPKA HVEAPIAGSM VLAAVLLKLG GYGIIRIISI   250
Tste          201 MLAFLVKMPL YGTHLWLPKA HVEAPIAGSM ILAAVLLKLG GYGIIRLMPT   250
Gvit          201 TLAFMVKMPL YGLHLWLPKA HVEAPIAGSM ILAAVLLKLG GYGIIRMTPM   250
Cvar          201 LLAFLVKMPL YGLHLWLPKA HVEAPIAGSM VLAATLLKLG GYGIIRITPI   250

                                260        270        280        290        300
Ttri_1        251 LPPTQTPRSL ITAPLTTPYP FIVLALWGAV MTSMICLRQP GLKSIITYSS   300
Ttri_2        251 LPPTQTPHNI ITAPLTTHYP FIVLTLWGAI MTSMICLRQP DLKSIIAYSS   300
Tste          251 VT-------- -NITQTLYHP FIVLALWGMI MTSMICLRQT DLKSIIAYSS   300
Gvit          251 LL-------- -HMTQTMYFP FIVLSLWGMI MTSLICLRQT DLKSIIAYSS   300
Cvar          251 LP-------- -PLTQTIYLP FITLALWGMI MTSLICLRQT DLKSIIAYSS   300

                                310        320        330        340        350
Ttri_1        301 ISNTGLVVAA ALIQTPWSIN SAMLLMITHD LTSSMLFCLA NTNYERTHSR   350
Ttri_2        301 ISHMGLVVAA ALIQTPWSIN GAMLLMIAHG LTSSMLFCLA NTNYERTHSR   350
Tste          301 VSHMGLVIAG TLIQTPWGTK GAMILMIAHG LTSSMLFCLA NTNYERTHTR   350
Gvit          301 VSHMGLVIAA TLINTPWSIA GAMTLMIAHG LTSSVLFCLA NTNYERMHTR   350
Cvar          301 VSHMGLVTAA ALIHTPWSIT GAMILMIAHG LTSSMLFCLA NTNYERTHSR   350

                                360        370        380        390        400
Ttri_1        351 TLILTRGMHL ALPLMATWWM MASMANMSLP PTINMLGKIT TISSAFSWNT   400
Ttri_2        351 TLVLTRGLQL ALPLMTTWWL TASLANMSLP PTINMLGEIM IISSTFNWST   400
Tste          351 TLILTRGLQL ALPLMTSWWM AASLTNLALP PTINLLGEIM IITSIFNWNT   400
Gvit          351 TLLLTRGLLF ALPLMTTWWL MASLMNMALP PTINLLAELM IITSLFNWNT   400
Cvar          351 TLLIARGLQL ILPLMTTWWL MASLANLALP PTINLMGELI IISTLFSWST   400

                                410        420        430        440        450
Ttri_1        401 TTSILMTLAT LLTTAYSLHF LITPLHNELS TLTKVPPTQT REHLLMTLHL   450
Ttri_2        401 MTIILTALTT LLTAAYSLYI FSTTQRSKLS TPTKLPPTQT REHLLMTLHL   450
Tste          401 ITIALTAMAT VLTATYSLHI FLTTQRHKLS TRTPLKPTHT REHLLMTLHL   450
Gvit          401 LTIILTGTTT LITATYSLYI FMSSQWSTLP PNKTLSMTHT REHLLLTLHL   450
Cvar          401 PTILITGLTT LITATYSLYI FLMTQRNKMM NHLTFPPSHT REHLLMSLHM   450

                                460        470        480        490        500
Ttri_1        451 APMAVFTFSP DLTPLTFDLI EEFYLWALGL G......... ..........   500
Ttri_2        451 TPMILLILSP NLVL...... .......... .......... ..........   500
Tste          451 APLMLLILHP NLML...... .......... .......... ..........   500
Gvit          451 IPLMALIINP NLMF...... .......... .......... ..........   500
Cvar          451 IPLTLLILTP KLMM...... .......... .......... ..........   500
```

Fig. S5. Alignment of ND4 amino acid sequences of geckos. Amino acid translation of ND4 gene sequences from Fig. S4 are compared and aligned. For taxon abbreviations, see the legend of Fig. S4.
