# Supplementary material for: Gene rearrangements in gekkonid mitochondrial genomes with shuffling, loss, and reassignment of tRNA genes
Source: BMC Genomics. 2014 Oct 24;15(1):930. doi: 10.1186/1471-2164-15-930 (PMC4223735; doi:10.1186/1471-2164-15-930)
Supplement: Supplementary file 3 — Additional file 3: Figure S7: 1% agarose gel electrophoresis of PCR products amplified from closely related gecko species using rND2-4L and H5934m. Figure S8. 1% agarose gel electrophoresis of PCR products amplified from closely related gecko species using rND3-1L and rCUN-3H. (PDF 141 KB) [file 12864_2014_6628_MOESM3_ESM.pdf]

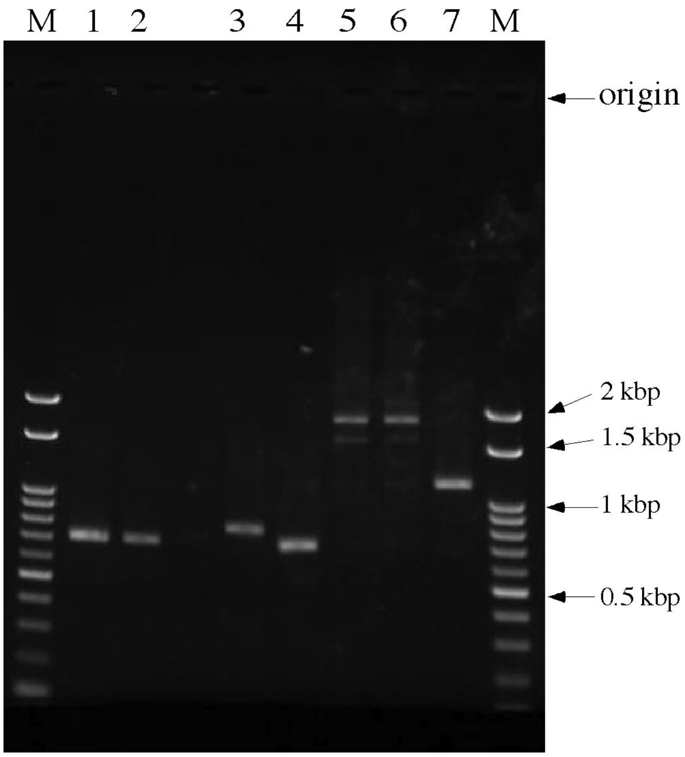

Fig. S7. 1% agarose gel electrophoresis of PCR products amplified from closely related gecko species using rND2-4L and H5934m. Lane 1: *Tropiocolotes tripolitanus*; lane 2: *Tropiocolotes steudneri*; lane 3: *Stenodactylus doriae*; lane 4: *Stenodactylus slevini*; lane 5: *Stenodactylus petrii* (individual No. 1 used for mitogenomic sequencing); lane 6: *Stenodactylus petrii* (individual No. 2); lane 7: *Stenodactylus petrii* (individual No. 3); and lane M: 100bp ladder size marker. The gel was stained with ethidium bromide.

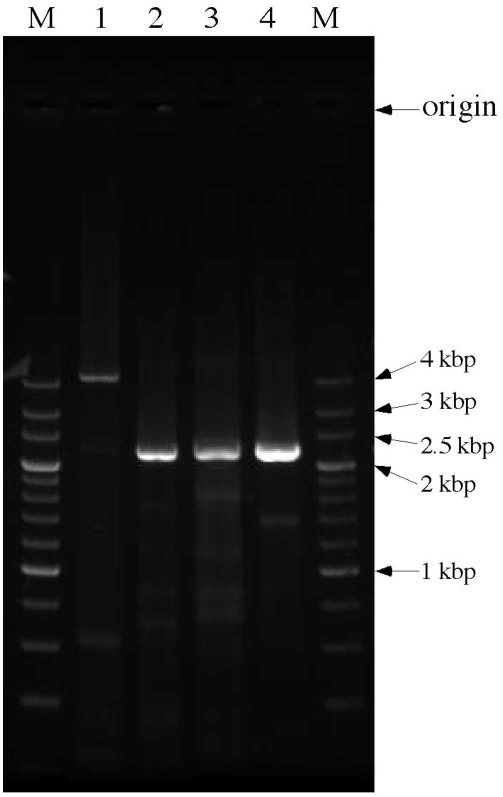

Fig. S8. 1% agarose gel electrophoresis of PCR products amplified from closely related gecko species using rND3-1L and rCUN-3H. Lane 1: *Tropiocolotes tripolitanus*; lane 2: *Microgecko persicus*; lane 3: *Tropiocolotes steudneri*; lane 4: *Stenodactylus petrii*; and lane M: 200bp ladder size marker. The gel was stained with ethidium bromide.
