# Supplementary material for: Gene rearrangements in gekkonid mitochondrial genomes with shuffling, loss, and reassignment of tRNA genes
Source: BMC Genomics. 2014 Oct 24;15(1):930. doi: 10.1186/1471-2164-15-930 (PMC4223735; doi:10.1186/1471-2164-15-930)
Supplement: Supplementary file 4 — Additional file 4: Table S2: Sequences determined from closely related species in this study. Table S3. Evidence for no genetic code change in glutamine and glutamic acid codons for four gecko species. (PDF 83 KB) [file 12864_2014_6628_MOESM4_ESM.pdf]

Table S2. Nucleotide sequences determined from related species in this study

Species	Region	Accession No.
Stenodactylus petrii (individual No. 2)	between ND2 and CO1 genes	AB935399
Stenodactylus petrii (individual No. 3)	between ND2 and CO1 genes	AB935400
Stenodactylus doriae	between ND2 and CO1 genes	AB935401
Stenodactylus slevini	between ND2 and CO1 genes	AB935402
Microgecko persicus	between ND2 and CO1 genes	AB935403
Uroplatus pietschmanni	between ND6 and cytb genes	AB926435
Uroplatus sikorae	between ND6 and cytb genes	AB926436
Uroplatus guentheri	between ND6 and cytb genes	AB926437
Uroplatus lineatus	between ND6 and cytb genes	AB926439
Uroplatus phantasticus	between ND6 and cytb genes	AB926438
Uroplatus pietschmanni	between trnP and trnF genes	AB935602
Uroplatus sikorae	between trnP and trnF genes	AB935604
Uroplatus guentheri	between trnP and trnF genes	AB935603
Uroplatus lineatus	between trnP and trnF genes	AB935606
Uroplatus phantasticus	between trnP and trnF genes	AB935605

Table S3. Evidence for no genetic code change in glutamine and glutamic acid codons for four gecko species

Taxon	Codons for Gln		Codons for Glu		Number of all codons
	CAA	CAG	GAA	GAG	
Tropiocolotes tripolitanus	67 (85.1/74.6)	13 (76.9/53.8)	55 (92.7/85.5)	18 (83.3/77.8)	3447
Tropiocolotes steudneri	75 (81.3/66.7)	10 (100.0/90.0)	61 (90.2/83.6)	11 (90.9/100.0)	3436
Uroplatus fimbriatus	64 (68.8/67.2)	18 (72.2/50.0)	55 (89.1/81.8)	18 (94.4/94.4)	3442
Uroplatus ebenaui	65 (73.8/67.7)	14 (71.4/64.3)	42 (90.5/81.0)	31 (80.6/80.6)	3441

Values in each cell are the number of the corresponding codons that appear in alignable regions of 13 protein-coding genes for each taxon.

Values in parentheses are percentages in which the corresponding codon positions in *Coleonyx variegatus* (left to the slash) and human (right to the slash) have an identical amino acid (i.e., Gln for CAA and CAG and Glu for GAA and GAG). The vertebrate mitochondrial genetic code was first established for human (Anderson et al., Nature 290: 457-465, 1981). *C. variegatus* is one of gecko species from which a complete mitogenome was sequenced with no evidence of genetic code alteration (Kumazawa, Gene 388: 19-26, 2007). High percentages in this table (50% or more) suggest no genetic code change for the four codons in each of the four gecko species (see, e.g., Himeno et al., Gene 56: 219-230, 1987).
